# The lncRNA *RZE1* Controls Cryptococcal Morphological Transition

**DOI:** 10.1371/journal.pgen.1005692

**Published:** 2015-11-20

**Authors:** Nadia Chacko, Youbao Zhao, Ence Yang, Linqi Wang, James J. Cai, Xiaorong Lin

**Affiliations:** 1 Department of Biology, Texas A&M University, College Station, Texas, United States of America; 2 Department of Veterinary Integrative Biosciences, Texas A&M University, College Station, Texas, United States of America; University College Dublin, IRELAND

## Abstract

In the fungal pathogen *Cryptococcus neoformans*, the switch from yeast to hypha is an important morphological process preceding the meiotic events during sexual development. Morphotype is also known to be associated with cryptococcal virulence potential. Previous studies identified the regulator Znf2 as a key decision maker for hypha formation and as an anti-virulence factor. By a forward genetic screen, we discovered that a long non-coding RNA (lncRNA) *RZE1* functions upstream of *ZNF2* in regulating yeast-to-hypha transition. We demonstrate that *RZE1* functions primarily *in cis* and less effectively *in trans*. Interestingly, *RZE1*’s function is restricted to its native nucleus. Accordingly, *RZE1* does not appear to directly affect Znf2 translation or the subcellular localization of Znf2 protein. Transcriptome analysis indicates that the loss of *RZE1* reduces the transcript level of *ZNF2* and Znf2’s prominent downstream targets. In addition, microscopic examination using single molecule fluorescent in situ hybridization (smFISH) indicates that the loss of *RZE1* increases the ratio of *ZNF2* transcripts in the nucleus *versus* those in the cytoplasm. Taken together, this lncRNA controls *Cryptococcus* yeast-to-hypha transition through regulating the key morphogenesis regulator Znf2. This is the first functional characterization of a lncRNA in a human fungal pathogen. Given the potential large number of lncRNAs in the genomes of *Cryptococcus* and other fungal pathogens, the findings implicate lncRNAs as an additional layer of genetic regulation during fungal development that may well contribute to the complexity in these “simple” eukaryotes.

## Author Summary

The involvement of non-protein regulators in developmental processes in higher eukaryotes is an area that has come to light more recently. Earlier known as the dark matter of the genome, the non-protein coding genes are now recognized for their important regulatory roles in the life of eukaryotes. Using forward genetic screen, we identified *RZE1* as a lncRNA with key function in regulating morphogenesis in *Cryptococcus*. We further discovered that *RZE1* regulates the transcription and transcript export of *ZNF2*, which encodes the key morphogenesis transcription factor. *RZE1* is the first functionally characterized lncRNA in a human fungal pathogen. Given the potential large number of lncRNAs in *Cryptococcus* and other fungal pathogens, the *RZE1*-*ZNF2* regulatory system could serve as a paradigm for the investigation of lncRNAs in development and virulence in eukaryotic pathogens.

## Introduction

In many human fungal pathogens, the morphological transition from yeast to hypha plays a central role in pathogenesis [1, 2], as demonstrated in the ascomycetes *Candida albicans*, *Penicillium marneffei*, *Histoplasma capsulatum*, *Coccidioides immitis*, and *Paracoccidiodides brasiliensis* [3–6]. Different morphotypes also display different levels of pathogenicity in the basidiomycetous fungus *Cryptococcus neoformans* [1, 7], the causative agent of the deadly cryptococcal meningitis [8]. Although primarily considered as yeasts, *Cryptococcus* undergoes yeast-to-hypha transition during unisexual mating (self-fruiting) or bisexual **a**-α mating [9–11].

**Fig 1:**
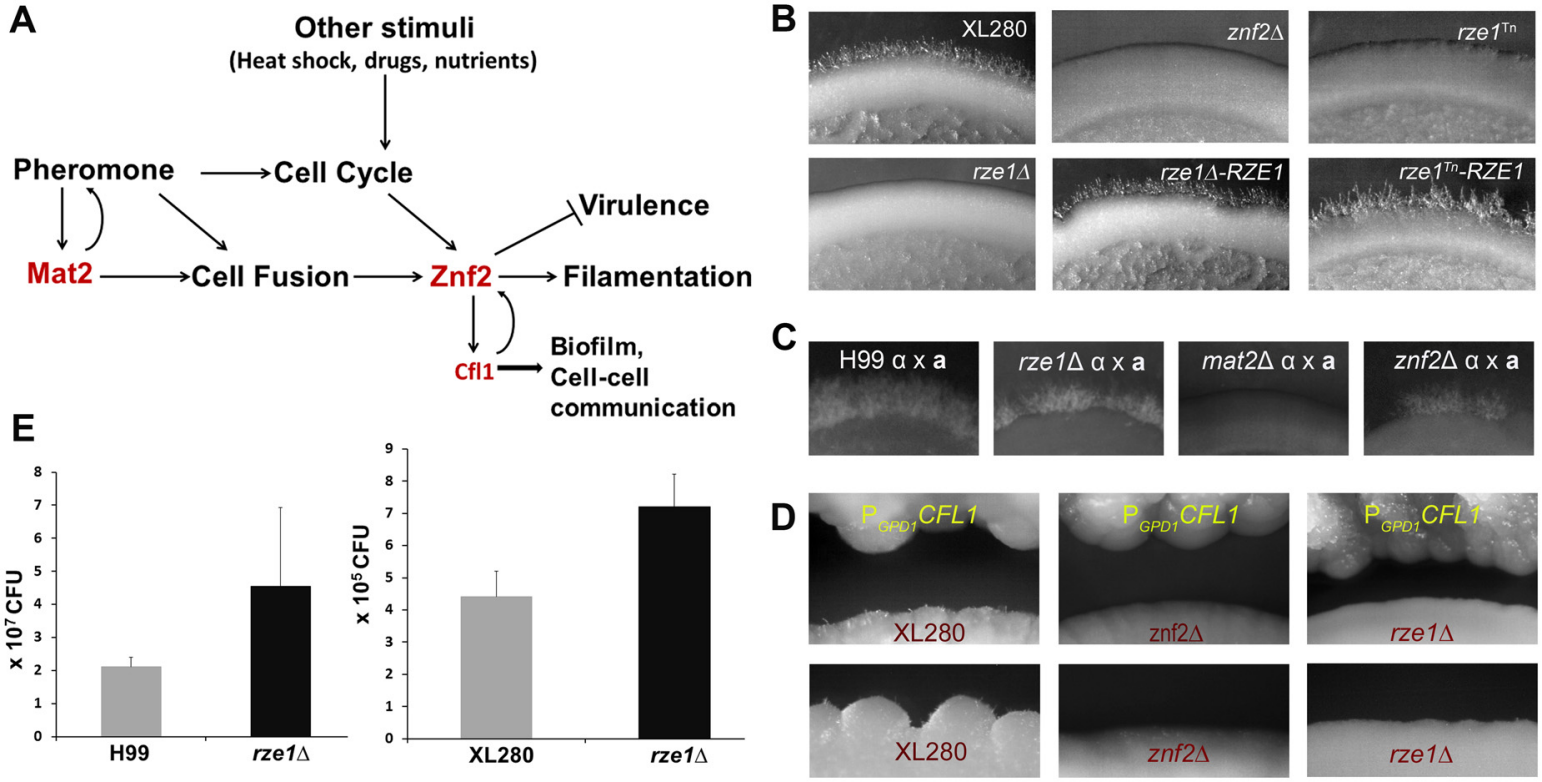
The phenotypes caused by the loss or disruption of *RZE1* resemble the ones caused by the deletion of *ZNF2*. (A) A diagram of various signals (pheromone and other stimuli) that lead to activation of *ZNF2* and consequently filamentation in *Cryptococcus*. (B) The self-filamentation assay of the wild type XL280, the *znf2*Δ mutant, the *rze1* ^Tn^ mutant, the *rze1*Δ mutant, and the complemented strains *rze1* ^Tn^-pRZE1 and *rze1*Δ-p*RZE1* with the wild type allele of *RZE1* integrated at the native locus. Strains were cultured on V8 medium for 4 days. (C) Filamentation assay of unilateral crosses during bisexual mating involving a mutant strain and a wild-type mating partner. The α partner is a mutant in the non-self-filamentous H99 background (the *rze1*Δ mutant, the *mat2*Δ mutant, and the *znf2*Δ mutant). The **a** partner is the wild type KN99**a** that is congenic with H99. Cells were cultured on V8 medium for about 2 weeks. (D) Confrontation assay with the P_*GPD1*_-*CFL1* strain as the donor and the wild type XL280, the *rze1*Δ mutant, or the *znf2*Δ mutant as the recipient. (E) Fungal burden analysis from lungs of mice inoculated with the *rze1*Δ mutants and the corresponding wild-type strains. *p* = 0.0429 for H99 background and 0.0716 for XL280 background.

The zinc finger transcription factor Znf2 ultimately controls this morphotype transition. During mating, Znf2 is activated by the pheromone MAPK pathway controlled by the HMG domain transcription factor Mat2 [12–15] (Fig 1A). Mat2 is essential for pheromone sensing and response, which leads to the cell fusion event. Hyphal growth commences after cell fusion and eventually gives rise to fruiting structures and meiotic spores [9, 16]. However, Mat2 does not control hyphal morphogenesis *per se* [12]. By contrast, Znf2 governs hypha generation and it is dispensable for the early mating events like cell fusion [12, 17] (Fig 1A). Under non-mating inducing conditions, Znf2 could be activated by the matri-cellular signal protein Cfl1 through a positive feedback regulation [18, 19]. It is unknown whether other host or environmental factors can also regulate Znf2 activity.

Znf2 is an anti-virulence factor in the mouse model of cryptococcosis [12, 20]. The deletion of the *ZNF2* gene locks the fungal cells in the yeast form, making them more virulent [12]. Conversely, the activation of *ZNF2* drives filamentation and attenuates *Cryptococcus* virulence [21, 22]. The *ZNF2* overexpression cells, either in the live or heat-killed form, can protect the hosts from a subsequent challenge with otherwise lethal wild-type cells [22]. Thus manipulation of *ZNF2* activity could be a potential means to alleviate cryptococcosis. Besides its anti-virulence effect during cryptococcal infection in a mammalian host, Znf2 also shapes cryptococcal interaction with other heterologous hosts, such as the soil amoeba *Acanthamoeba castellanii* and the insect *Galleria mellonella* [23]. The essential role of Znf2 in *Cryptococcus* sexual cycle and its pivotal role in regulating cryptococcal interaction with various host species make this transcription factor a potential target for multi-layered regulation in response to various stimuli.

To identify the upstream regulators of *ZNF2*, we conducted a forward genetic screen to find mutations that cause similar phenotypes as those caused by the disruption of *ZNF2*. The screen led to the discovery of *RZE1* that functions upstream of *ZNF2*. Further investigation revealed that *RZE1* functions primarily as a *cis*-acting, and less efficiently a *trans*-acting, lncRNA. Furthermore, this lncRNA is functionally restricted to its native nuclei based on heterokaryon assay. We found that *RZE1* exerts its impact on cryptococcal morphogenesis by regulating *ZNF2* transcription and by influencing the nuclear *versus* cytoplasmic distribution of *ZNF2* transcripts, which consequently affects *ZNF2*’s ability to get translated into protein.

Although genomic and transcriptomic data suggest the presence of lncRNAs showing infection- or tissue-specific expression in fungal species pathogenic to plants and animals [24], it is unclear if lncRNAs have any biological relevance in the life cycle or the development of human fungal pathogens. *RZE1* is the first lncRNA that is functionally characterized in a human fungal pathogen. The importance of *RZE1* in cryptococcal morphogenesis raises the possibility that lncRNAs may be important regulators that contribute to the complexity in genetic regulation in these eukaryotic pathogens.

## Results

### Identification of the RZE1 gene for its importance in filamentation via forward genetic screen

*Cryptococcus* undergoes filamentation in response to the mating signal and other environmental cues. Znf2 is the essential regulator of this morphological switch and it bridges morphogenesis and virulence in this fungal pathogen [12, 17–19]. To identify the regulatory network of the Znf2-controlled filamentation pathway, we conducted a random insertional mutagenesis screen for *znf2*Δ-like phenotype in the self-filamentous strain XL280 [25]. XL280 has been commonly used in morphogenesis studies [12, 20, 25]. It has a publically released genome sequence (~19 Mb, ~7000 genes) [26] and a well characterized congenic pair [21]. We generated 63,000 insertional mutants *via Agrobacterium*-mediated transformation in XL280 and screened these mutants on filamentation-inducing V8 medium for non-filamentous phenotypes. Among a set of selected non-filamentous mutants, 15 had their insertion site identified by inverse PCR and sequencing (Table 1). Of the 15 insertion sites identified, one T-DNA insertion (Tn) in mutant X261 was found to be in the genetic locus that we named *RZE1*. The *RZE1* gene encodes a 1,268 nt long transcript in XL280 based on our primer walking and RACE PCR results (S1 Fig). Only one transcription start site and one transcription stop site were identified for *RZE1* under the tested condition.

**Table 1:** Insertion mutants that show lack of filamentation and their insertion sites.

**Strain name**	**Locus name in JEC21**	**Locus name in H99**	**Gene name**
**X15**	CNI00570	CNAG_04500	Hypothetical protein
**X207**	CNB04320	CNAG_03945	Unknown protein with regulatory subunit of the histone H2A phosphatase complex
**X261**	Upstream of CNG02160	Upstream of CNAG_03366	*RZE1*, Upstream of Zinc finger transcription factor *ZNF2*
**X318**	CNC02270	CNAG_01723	Hypothetical protein
**X319**	CNH01180	CNAG_05456	Hypothetical protein
**X322**	CNF04180	CNAG_06601	Amidohydrolase
**X331**	CNK00830	CNAG_02597	Hypothetical protein
**X333**	CNI00570	CNAG_04500	Hypothetical protein
**X336**	CNH00730 and neighbors	CNAG_05405	Hypothetical protein
**X337**	CNF02530	CNAG_05721	Multi-functional beta oxidation protein
**X343**	CNB03700	CNAG_07483	DNA polymerase zeta subunit
**X351**	CNG03090	CNAG_03266	Spliceosome assembly related protein
**X412**	CNF04390	CNAG_07989	Hypothetical protein in velvet superfamily
**X413**	CNI00560	CNAG_04501	Anthranilate synthase
**X419**	CNF00330	CNAG_07695	Gama-aminobutyric acid transporter

### Disruption of RZE1 recapitulates the phenotypes caused by the deletion of ZNF2

The *rze1* ^Tn^ mutant, like the *znf2*Δ mutant, is non-filamentous under mating-inducing conditions such as on V8 media (Fig 1B). To confirm the role of *RZE1* in hyphal growth, we deleted the *RZE1* gene in the XL280 background. The targeted deletion of *RZE1* also abolished self-filamentation (Fig 1B). To ensure that the non-filamentous phenotype of the *rze1* ^Tn^ and the *rze1*Δ mutants was attributable to the disruption of *RZE1* and not due to other cryptic mutations, a wild-type copy of the *RZE1* gene was re-introduced ectopically into the *rze1* ^Tn^ and the *rze1*Δ mutants. The ectopically integrated *RZE1* partially restored the filamentation defects in these *rze1* mutants (S2 Fig). We also introduced the wild-type *RZE1* back to its native locus in the *rze1* ^Tn^ and the *rze1*Δ mutants. The introduced *RZE1* gene at its native locus effectively restored the ability of both mutants to filament (Fig 1B), indicating that *RZE1* indeed is required for filamentation.

The pheromone sensing pathway is critical for filamentation under mating-inducing conditions. Disruption of the pheromone sensing pathway (e.g. the deletion of HMG domain transcription factor Mat2 or the MAPKK Ste7), even in one mating partner, will abolish filamentation produced by bisexual mating between **a** and α partners [12, 14]. The lack of the bisexual mating hyphae is due to lack of cell fusion. It is previously established that the disruption of *ZNF2* does not impair pheromone pathway or abolish cell fusion [12]. Thus a unilateral cross involving the *znf2*Δ mutant and a wild-type partner still produces mating filaments, in contrast to the unilateral cross involving one *mat2*Δ mating partner [12]. We then decided to assess if this also holds true for *RZE1*. To avoid the complication due to self-filamentation in the XL280 background, we chose to delete *RZE1* in the non-self-filamentous strain H99. In the H99 background, hyphae can only result from bisexual mating following cell fusion between **a** and α cells activated by the pheromone pathway. We found that the unilateral crosses between the *rze1*Δ (or the *rze1* ^Tn^) mutant with a wild-type mating partner produced mating filaments (Fig 1C), as in the *znf2*Δ mutant and unlike the *mat2*Δ mutant (Fig 1C). Consistently, the pheromone production in the *rze1*Δ mutant was at par with the wild type, indicating that *RZE1*, like *ZNF2*, is not required for pheromone sensing pathway.

Under non-mating inducing conditions like on the YPD medium, *ZNF2* and thereby filamentation can be activated by the matricellular signaling protein Cfl1 through a positive feedback loop [18]. Znf2 is absolutely necessary for *Cryptococcus* to respond to exogenous Cfl1 signal to promote filamentation [20]. Similarly, we found that *RZE1* is also required for the recipient strain to filament in response to the *CFL1* protein released from nearby donor cells under mating-suppressing conditions (Fig 1D).

Morphological switch from the yeast state to the filamentous state is inversely correlated with virulence in *Cryptococcus*. Consequently, the loss of *ZNF2* modestly increases fungal virulence [20]. Thus we hypothesize that the upstream regulator of *ZNF2* might also influence cryptococcal virulence potential. Since mice are highly susceptible to XL280 (*C*. *neoformans* var. *neoformans*, serotype D) [21, 27] and H99 (*C*. *neoformans* var. *grubii*, serotype A) [28, 29], we decided to compare the virulence between the wild-type strain and the *rze1*Δ mutant made in both XL280 and H99 backgrounds using the fungal burden assays. We inoculated the mice with the *rze1*Δ mutants and their corresponding wild-type strains through inhalation and measured the fungal burdens in the lungs at day 10 post inoculation. The fungal burdens in the lungs infected by the *rze1*Δ mutants were close to 2 fold higher than those infected by the corresponding wild-type controls (Fig 1E), indicating enhanced virulence of the *rze1*Δ mutants. Given that an increase in virulence with gene disruption is rare, these results are consistent with the observed enhanced virulence of the *znf2*Δ mutant, which showed about 2.4 fold increase in lung fungal burden compared to the wild type at the same time point during infection [12, 20]. In line with our previous observations for the *znf2*Δ mutant [12, 20], the *rze1*Δ mutant did not show any apparent difference from the wild type in classic virulence traits such as melanization, capsule production, or thermo-tolerance (S3 Fig). We also did not observe any apparent alteration in the susceptibility of the *rze1*Δ mutant to stressors like SDS, caspofungin (inhibitor of β -1,3-glucan synthase), calcoflour white (inhibitor of chitin), iron chelator, UV radiation, or oxidative stress (H_2_O_2_) when compared to the wild-type control (S3 Fig). Taken together, these observations indicate that the disruption of *RZE1* recapitulates *in vitro* and *in vivo* phenotypes caused by the deletion of *ZNF2*. Thus *RZE1* might be a highly selective regulator of *ZNF2* or a major target of *ZNF2*.

### *RZE1* is physically and functionally upstream of ZNF2

**Fig 2:**
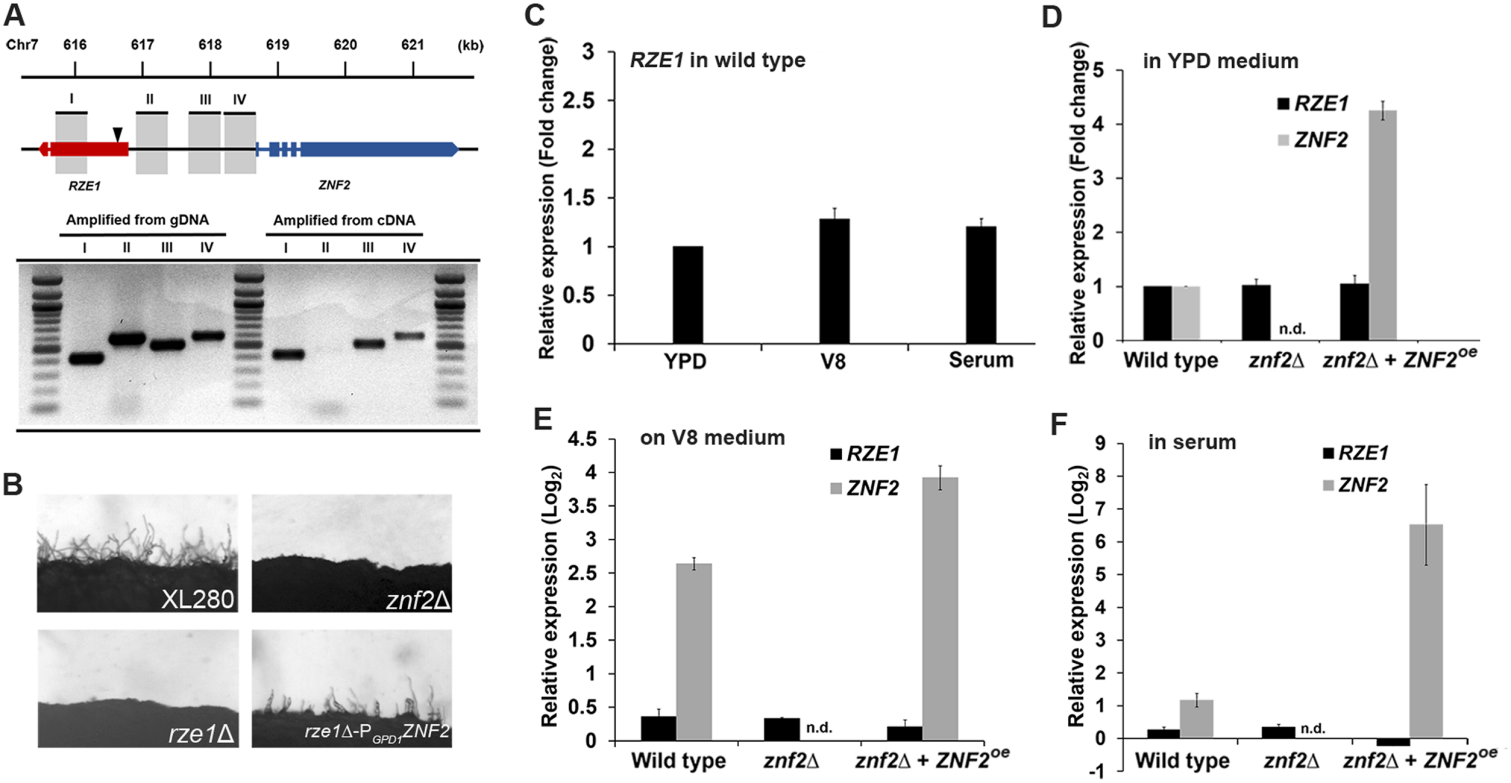
*RZE1* is an independent gene and it functions upstream of *ZNF*. (A) The diagram of the *RZE1-ZNF2* genomic region in XL280. The location of the insertion site in the *rze1* ^Tn^ mutant identified by inverse PCR is shown by the arrow. The products amplified from different regions between *RZE1* and *ZNF2* using either cDNA or genomic DNA as the template were separated on a gel by electrophoresis. The introns of the *ZNF2* gene and one intron of the *RZE1* gene are indicated as spaces. (B) The constitutive expression of *ZNF2* (P_*GPD1*_-*ZNF2*) in the *rze1*Δ mutant partially restores filamentation in the mutant. The *znf2*Δ mutant and the *rze1*Δ mutant in XL280 background are blocked in filamentation. (C) The relative transcript levels of *RZE1* in wild-type H99 cultured in YPD medium, on V8 agar medium during mating, or in fetal bovine serum for 24 hours as measured by qRT-PCR. The transcript level of *RZE1* in YPD medium was set as 1 for comparison. (D-F) The relative transcript levels of *RZE1* and *ZNF2* in the wild-type H99, the *znf2*Δ mutant, and the *ZNF2* ^oe^ strain when cultured in YPD medium (D), on V8 agar medium during mating (E), or in fetal bovine serum for 24 hours (F) as measured by qRT-PCR. n.d.: not detectable. For panels D-F, the transcript level of *RZE1* or *ZNF2* of the wild type in YPD medium was set arbitrarily as 1 for comparison (1 in fold change or 0 in Log_2_ value).

*RZE1* is located ~2.5 kb upstream of *ZNF2*. Although the average intergenic space is less than 800 bp in *Cryptococcus* [30], the physical location of *RZE1* raises a concern that the *RZE1* transcript could be part of the *ZNF2* transcript and the disruption of *RZE1* would cause the disruption of *ZNF2* itself. To address this concern, we amplified and compared four regions (I—IV in Fig 2A) that cover *RZE1*, the beginning of the *ZNF2* coding region, and the region between *RZE1* and *ZNF2*, using templates of total cDNA or genomic DNA derived from the wild-type XL280 strain. Region II that lies between *RZE1* and the 5’ region upstream of the *ZNF2* ORF failed to yield any detectable amplicon when cDNA was used as the template (Fig 2A). This suggests that *RZE1* is likely produced as a separate transcript independent from *ZNF2*. Furthermore, the ability of the ectopically introduced *RZE1* to partially complement the *rze1* ^Tn^ and the *rze1*Δ mutants (S2A Fig) reinforces the idea that *RZE1* is a functionally independent transcript from that of *ZNF2*. In addition, since the phenotype of the *znf2*Δ mutant can be complemented effectively using an ectopic copy of *ZNF2* with 1kb sequence upstream of the *ZNF2* ORF that does not include *RZE1* (S4 Fig), it is reasonable to conclude that *RZE1* and *ZNF2* encode separate transcripts that can function independently.

To investigate the relationship between *RZE1* and *ZNF2*, we examined the *RZE1* transcript level in the wild type, the *znf2*Δ mutant, and the *ZNF2* ^oe^ strain under three different growth conditions (YPD/rich medium, serum/host relevant, and V8/mating-inducing). The transcript level of *RZE1* in the wild-type H99 strain was relatively stable, and there was no dramatic difference when cells were cultured in YPD medium, on V8 medium, or in serum (Fig 2C). Under the same culture conditions, the *RZE1* transcript level remained constant among the different strains (black bars in Fig 2D–2F), even though the *ZNF2* transcript level was drastically different among these strains (grey bars in Fig 2D–2F). This indicates that *RZE1* is not responsive to changes in the *ZNF2* transcript level. This result supports the earlier conclusion that *RZE1* is a transcript independent of *ZNF2* and likely functions in the filamentation pathway upstream of *ZNF2*. Consistently, the constitutive expression of *ZNF2* driven by the *GPD1* promoter led to the partial restoration of filamentation to these *rze1* mutants (Fig 2B), indicating that *ZNF2* indeed functions downstream of *RZE1*. These observations are in accordance with the idea that *RZE1* functions in the filamentation pathway upstream of *ZNF2*.

### *RZE1* likely functions as a lncRNA

**Fig 3:**
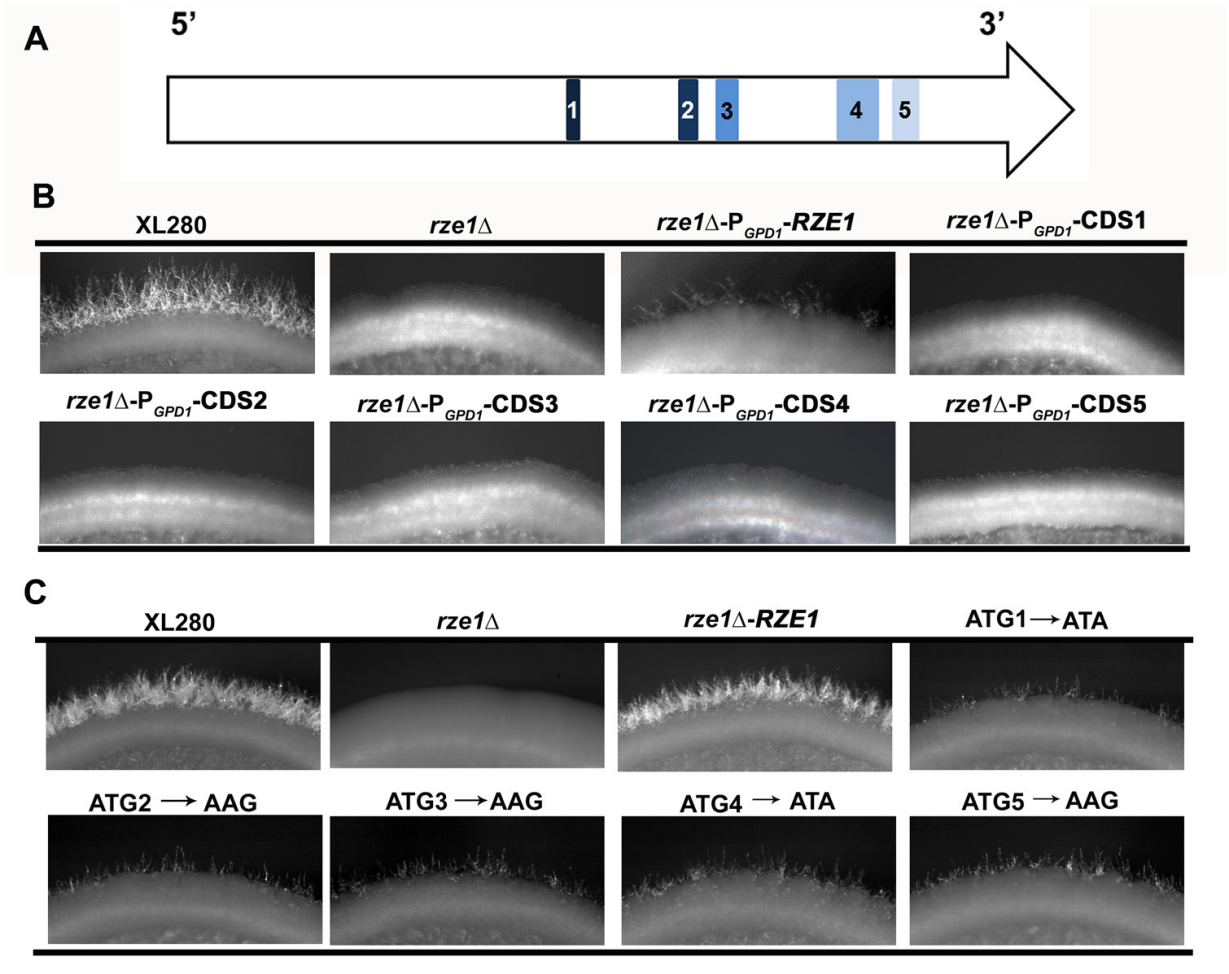
*RZE1* is a likely lncRNA. (A) The diagram of the five putative small open reading frames in *RZE1* is shown in the top row. (B) The constitutive (P_*GPD1*_) or overexpression (P_*CTR4-2*_, see S5 Fig) of each putative open reading frame of *RZE1* does not restore filamentation in the *rze1*Δ mutant. Cells were cultured on V8 medium for 4 days. (C) Partial complementation of the *rze1*Δ mutant with *RZE1* modified for each potential translation start codon. All the start codon mutated alleles generated by single nucleotide mutagenesis and the wild-type allele of *RZE1* were integrated at its native genetic locus in the *rze1*Δ mutant background. The mutated alleles showed partial complementation. Better complementation was achieved by the wild-type allele of *RZE1*. Cells were cultured on V8 medium for 4 days.

Based on the *Cryptococcus* EST databases, the *RZE1* transcript is present in all subspecies of the *Cryptococcus neoformans* species complex, namely *C*. *neoformans* var. *grubii* (serotype A), *C*. *neoformans* var. *neoformans* (serotype D), and the subspecies *C*. *gatii* (serotype B and C). An analysis of the *RZE1* transcript sequence suggests that the *RZE1* gene is not a typical protein-coding gene. Rather than encoding one open reading frame (ORF), as expected for most mRNAs in fungi, *RZE1* contains 5 short potential ORFs (Fig 3A), with three potential translation start codons (ATG) in a poor translation context (S1 Fig). BLAST searches using these potential ORFs against fungal genome databases or GenBank did not yield any significant hits. Thus, these potential ORFs do not encode conserved protein products.

To test if products from any of the small ORFs confer *RZE1* function in filamentation, we inserted the constitutive active *GPD1* promoter [31] (Fig 3B) or the *CTR4* inducible promoter [32] (S5 Fig) upstream of each of the five ORFs contained within the *RZE1* transcript. We introduced these constructs into the *rze1*Δ mutant and then tested the transformants for their ability to filament. None of the P_*GPD1*_-ORF transformants were able to produce filaments (Fig 3B). Similarly, none of the P_*CTR4-2*_-ORF transformants were able to filament under inducing conditions (S5 Fig). The results suggest that the potential ORFs carried within the *RZE1* transcript are unlikely to produce protein products that function in the filamentation pathway. We postulate that *RZE1* may function as a lncRNA instead.

To further determine whether *RZE1* functions as a protein or a transcript, we did site-directed single nucleotide mutagenesis to alter the translation start codon of each of the five potential ORFs in *RZE1* (Fig 3C). We mutated ATG to ATA or AAG as such changes are known to almost abolish translation initiation in other fungi [33]. These codon changes are expected to prevent or considerably reduce the translation of these potential ORFs. We then introduced each of these mutated *RZE1* alleles into the *rze1*Δ mutant and selected transformants with the mutated *RZE1* allele integrated into the *RZE1*’s native locus. We then examined the ability of these transformants to produce hyphae. To our amazement, all the mutated *RZE1* alleles were able to restore the filamentation defect of the *rze1*Δ mutant (Fig 3C), although to a lesser degree compared to the wild-type *RZE1* allele integrated at the native locus (Fig 3C). This finding indicates that these *RZE1* alleles are at least partly functional in promoting filamentation, even though the specific nucleotides that are potential start-codons are mutated. Collectively, our results strongly suggest that *RZE1* is a long non-coding RNA regulating morphogenesis in *Cryptococcus*.

### *RZE1* is functionally restricted to the nucleus

**Fig 4:**
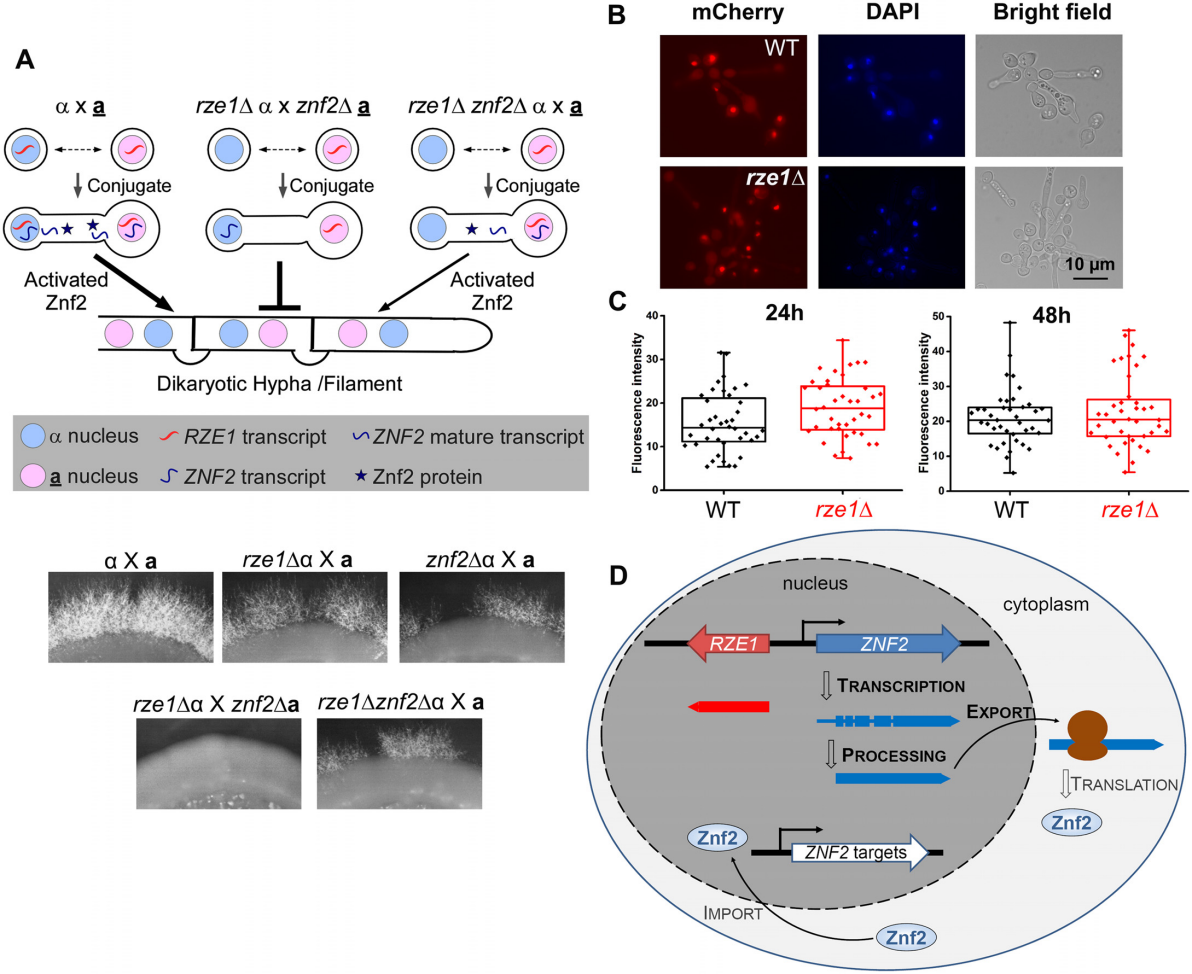
*RZE1* is functionally restricted to its native nucleus. (A) Heterokaryon assay showing the functional restriction of *RZE1* to the nucleus of its origin. The diagram above shows the rationale behind this heterokaryon assay. The strains used for this assay are all in the non-self-filamentous H99 background. The complete lack of filamentation in the *rze1*Δ α x *znf2*Δ **a** cross compared to the reduced filamentation from the *rze1*Δ*znf2*Δ α x **a** cross (gene dosage consideration) suggests that the *RZE1* from the second mating partner is unable to compensate the loss of *RZE1* from the first mating partner. Bisexual mating was assayed on V8 medium for about 3 weeks. The localization (B) and intensity (C) of the fluorescence signal from the P_*CTR4-2*_-Znf2-mCherry strain cultured in YPD+BCS with or without *RZE1*. *p* = 0.89 for 24h and 0.59 for 48h. (D) Working model for *RZE1*’s nuclear function in *Cryptococcus*.

As a lncRNA, it is possible to be functionally restricted to its target within its native nucleus, as *XIST* or *MALAT-1* [34–37], or to be functional in the cytoplasm [38]. This is in contrast to protein-coding mRNAs, which need to be exported from the nucleus to the cytoplasm for translation to make the functional products. Proteins produced by mRNAs would be able to function with its targets produced by any nucleus that shares the same cytoplasm. To narrow down the potential mode of action of *RZE1*, we decided to first examine if *RZE1* is functionally restricted to its native nucleus. For this purpose, we designed a heterokaryon assay where two cells conjugate and thus share the cytoplasm, but their nuclei do not fuse. Heterokaryon assay was previously used to show that the lncRNA *MALAT1* is functionally restricted to the nucleus [39]. Forming heterokaryons is a natural process during the **a**-α bisexual mating in *Cryptococcus* [9, 40, 41] (Fig 4A). The formation of α-**a** dikaryon after mating conjugation brings the homeodomain proteins Sxi1α and Sxi2**a** together [42], which triggers the expression of *ZNF2* and the production of dikaryotic mating hyphae [9, 12, 43]. It is important to note that *RZE1* controls filamentation but not conjugation (cell fusion), as established previously for Znf2 (Fig 1C) [12, 17]. Furthermore, the dikaryon will be blocked from filamentation only when *ZNF2* is disrupted in both mating partners [12, 43]. For this assay, we used cryptococcal strains in H99 background because H99 does not self-filament. As a result, filaments produced from the α-**a** bisexual mating are all derived from conjugated dikaryons. This allows us to examine if *RZE1* produced by one nucleus could compensate the loss of *RZE1* in the other nucleus that shares the same cytoplasm. The idea is that if the *RZE1* from one nucleus was able to function in the cytoplasm or in another nucleus in the same conjugated cell, then it would have been able to regulate *ZNF2* activity even if *ZNF2* is produced by the other nucleus (Fig 4A).

As expected, the unilateral crosses *rze1*Δ α x **a**, *znf2*Δ α x **a**, and *rze1*Δ*znf2*Δ α x **a** all filamented at a reduced level compared to the WT cross α x **a** (Fig 4A). However, the dikaryons generated from the cross *rze1*Δ α x *znf2*Δ **a** or the cross *rze1*Δ **a** x *znf2*Δ α failed to filament (Fig 4A). These dikaryons contain one nucleus with *RZE1* but no *ZNF2*, and the other nucleus with *ZNF2* but no *RZE1*. The inability of these dikaryons to filament indicates that *RZE1* produced by one nucleus failed to support the activity of *ZNF2* transcribed by the other nucleus even though the two nuclei shared the same cytoplasm. Because the diploid α/**a** wild-type cells in H99 background did not filament under this condition, it precludes us from further assessing whether the diploid cells derived from nuclear fusion of the *rze1*Δ**a** x *znf2*Δα heterokaryon could filament. The decreased ability of the heterozygous diploid cells (α/**a**) compared to the corresponding dikaryons (α-**a**) to continue through the mating program appears to be a common phenomenon in basidiomycetes. Nonetheless, these findings indicate that *RZE1* is functionally restricted to its native nucleus and as such it is highly unlikely for *RZE1* to directly affect the *ZNF2* translation or other post-translational processes that occur in the cytosol.

To further test the hypothesis that *RZE1* does not play a direct role in translation or Znf2 protein localization to the nucleus, we used a system where the expression of an ectopically introduced *ZNF2-mCherry* is controlled by the *CTR4* promoter. We compared the fluorescent intensity (indicative of protein level) and the subcellular localization of Znf2 in the presence or absence of *RZE1*. We found that the loss of *RZE1* does not affect either the intensity or the localization of Znf2-mCherry (Fig 4B and 4C). This is consistent with the idea that *RZE1* does not directly regulate *ZNF2* at the level of translation or protein translocation. Taken together, these results strongly suggest that *RZE1* is functionally restricted in its native nucleus, and is not directly involved in the Znf2 protein processing or translocation. In the nucleus, however, it is possible that *RZE1* could directly or indirectly regulate *ZNF2* at different levels (Fig 4D): (**i**) promote *ZNF2* transcription or transcript stability, (**ii**) support the production of the functional *ZNF2* transcript isoform through its influence on alternative start, alternative termination, or alternative splicing [28], or (**iii**) assist the export of the *ZNF2* transcript from nucleus to cytosol. Regulatory activity of *RZE1* in any of the above processes could result in an effect on the Znf2 protein level or activity in the wild type strain.

### *RZE1* regulates the *ZNF2* transcript level

**Fig 5:**
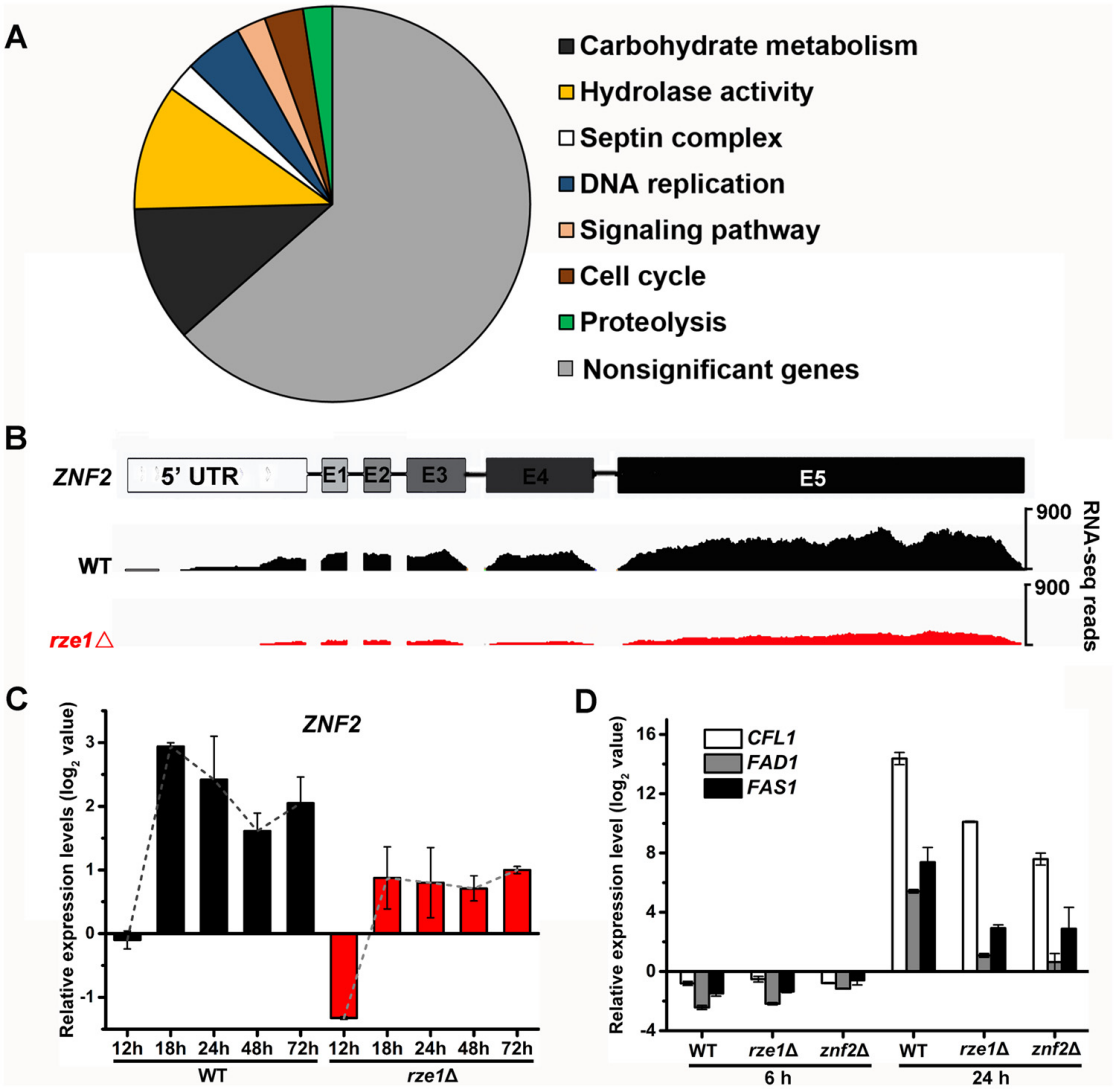
*RZE1* regulates the transcription of *ZNF2*. (A) The diagrammatic representation of enriched GO term classification of differentially expressed genes in the *rze1*Δ mutant compared to the wild-type strain. (B) The RNA sequence reads over the *ZNF2* locus is reduced ~2.6 fold in the *rze1*Δ mutant during self-filamentation at 24h on V8 medium (RNA-seq). (C) The transcript level of *ZNF2* is reduced in the *rze1*Δ mutant during self-filamentation at 12h, 18h, 24h, 48h, and 72h on V8 medium measured by qPCR. The transcript levels were compared to the transcript level of *ZNF2* in the wild type strain at the 12h time point. (D) The transcript levels of the downstream targets of *ZNF2*, namely *CFL1*, *FAD1*, and *FAS1*, were much reduced in the *rze1*Δ mutant and the *znf2*Δ mutant compared to those in the wild type.

The evidence presented so far suggests that *RZE1* functions in its native nucleus and it functions in a regulatory capacity upstream of *ZNF2* (Fig 4D). We decided to examine the first hypothesis of transcriptional control. To obtain a holistic view of the effect of the *RZE1* deletion on cryptococcal transcriptome, we first conducted a pilot comparative transcriptome analysis between the wild-type XL280 and the *rze1*Δ mutant undergoing self-filamentation by RNA sequencing (RNA-seq). A total of 265 genes with the 1.5 fold change as the cut-off threshold (*P*<0.05) were differentially expressed in the *rze1*Δ mutant relative to the wild type at 24h post inoculation on filamentation-inducing V8 media (S1 Table). These genes are classified into eight functional categories (Fig 5A). Among the enriched GO terms, genes involved in the septin complex, cell cycle, and DNA replication are known to affect morphogenesis [44–46], which is consistent with the regulatory function of *RZE1* in morphogenesis. Based on the RNA-seq data, the transcript level of *ZNF2* in the *rze1*Δ mutant was lower compared to the wild type at 24 hours post inoculation (Fig 5B). The reduction of the transcript level of *ZNF2* in the *rze1*Δ mutant was less obvious at 72 hours post inoculation compared to the 24h and 28h time points (S6B Fig). The lower *ZNF2* transcript level in the *rze1*Δ mutant was further confirmed by quantitative real-time PCR (Fig 5C). These results suggest that *RZE1*, directly or indirectly, up-regulates *ZNF2* at the transcriptional level.

Since filamentation is obvious at 24h in XL280 under inducing condition, we suspect that the *ZNF2* gene expression might be induced earlier. We therefore expanded our examination of the *ZNF2* transcript level in the *rze1*Δ mutant and the wild type to include more time points (12h, 18h, 24h, 48h, and 72h post inoculation on V8 medium) by qPCR. As expected [12], the *ZNF2* transcript level in the wild-type strain of all the later time points examined was increased compared to the reference level at 12h (Fig 5C). To our surprise, the *ZNF2* transcript level in the *rze1*Δ mutant is also increased, suggesting that the signal transduction to promote filamentation still occurs in the mutant. However, the level of *ZNF2* transcripts in the *rze1*Δ mutant is lower than that in the wild type, with the biggest difference observed at 18h (~4 fold) and smaller differences at 48h and 72h (Fig 5C). This might be consistent with the observation that the number of differentially expressed genes in the *rze1*Δ mutant is much higher at the 24h time point than the later 48h and 72h time points based on RNA-seq (S1 Table). These observations suggest an intriguing possibility that *RZE1* might play an important regulatory role in driving filamentation at the early stage of morphogenesis.

Given the modest effect on the *ZNF2* transcript level by the disruption of *RZE1*, we were surprised by the blocked filamentation in the *rze1*Δ mutant. Other mutants with reduced levels of *ZNF2* typically show reduced but not abolished filamentation. Thus it is enigmatic that the disruption of *RZE1* could completely abolish self-filamentation. The paradox between the observed reduction in the transcript level of *ZNF2* and the abolished filamentation in the *rze1*Δ mutant thus prompted us to examine the effect of *RZE1* deletion on the previously characterized downstream targets of Znf2 that are known to be important for filamentation: *CFL1*, *FAD1*, and *FAS1* [17, 18]. Both qPCR and RNA-seq results indicated that the transcript levels of these filamentation markers were considerably reduced in the *rze1*Δ mutant (Fig 5D and S7 Fig), often more comparable to those observed in the *znf2*Δ mutant (Fig 5D). The drastic reduction in the transcript level of *CFL1*, an auto-inducer working in positive feedback with *ZNF2* [18], is also in accordance with the results of the confrontation assay (Fig 1D). Thus it appears that although the deletion of *RZE1* only exerts a modest impact on the transcript level of *ZNF2*, it exerts a drastic impact on factors downstream of *ZNF2* and may inactivate at least part of the *ZNF2* regulon that controls filamentation. This raises the possibility that *RZE1* may affect additional processes between the *ZNF2* transcription and translation.

### The deletion of *RZE1* shows no obvious effect on *ZNF2* transcript intron splicing

**Fig 6:**
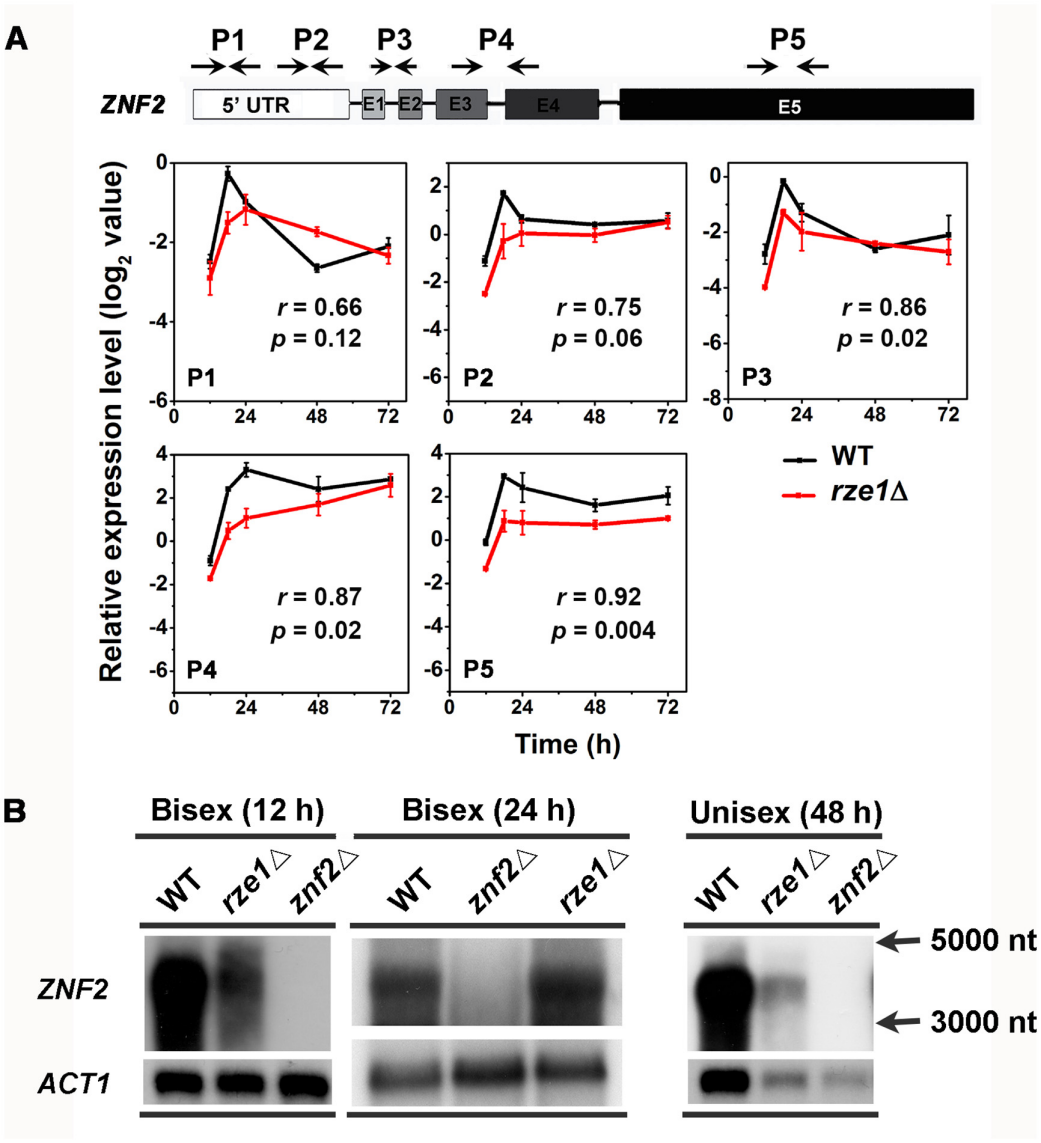
*ZNF2* transcript appears to be processed correctly in the absence of *RZE1*. (A) There was no apparent difference in the processed *ZNF2* transcripts in the *rze1*Δ mutant compared to those produced in the wild-type XL280 under the conditions analyzed. The level of different regions of *ZNF2* transcripts in the wild type and in the *rze1*Δ mutant cultured on V8 medium at various time points were analyzed by qPCR. The transcript level of *ZNF2* in the *rze1*Δ mutant shows a similar pattern in all regions. (B) Northern blot of Poly(A) RNAs probed with the DNA sequence of the *ZNF2* ORF showed the *ZNF2* transcripts of the same size in both the wild type and the *rze1*Δ mutant during bisexual mating (12h and 24h time points) and unisexual mating (48h time point). The *ACT1* transcripts serve as a control. The single stranded RNA markers of 5,000 nt and 3,000 nt were indicated to the right.

As transcript isoforms different from those in the wild type could be non-functional, we decided to examine the impact of *RZE1*’s disruption on *ZNF2* isoforms (Fig 4D). In *C*. *neoformans* 99.5% of all expressed genes have introns and an average gene contains multiple introns [30]. Thus alternative splicing could be one major way of generating different transcript isoforms. Indeed, multiple introns were found in *ZNF2* wild type allele in the serotype D strain XL280 (Fig 5B and S6A Fig) as well as in the serotype A strain H99 [28]. The RNA-seq data showed that introns of the *ZNF2* transcripts were spliced in both the wild-type strain XL280 and in the *rze1*Δ mutant (Fig 5B). Consistently, amplicons of the transcript that covered the whole *ZNF2* ORF region in the wild type and in the *rze1*Δ mutant by reverse transcription PCR showed no apparent difference in size, again supporting the idea that introns of the *ZNF2* transcripts could be spliced in the absence of *RZE1*. To examine if there was any quantitative difference in splicing of the *ZNF2* transcript at different regions, we analyzed the transcript level of five different regions of the *ZNF2* transcripts in the wild type and in the *rze1*Δ mutant during self-filamentation. These five regions included two regions in the 5’UTR, the junction between exon 1 and 2, the junction between exon 3 and 4, and the exon 5 region (Fig 6A). The levels of all five tested regions of the *ZNF2* transcripts were lower in the *rze1*Δ mutant relative to those in the wild type, again with the biggest difference observed at 18h and smaller differences observed at 48h and 72h (Fig 6A). The pattern was similar to the earlier observation based on the conserved exon 5 region using the P5 primer set (Fig 5C). Collectively, the results suggest that *RZE1* does not affect intron splicing of the *ZNF2* transcripts.

To test if *RZE1* plays a role in other aspects of *ZNF2* transcript processing, we performed northern blot and probed with the *ZNF2* ORF. As *ZNF2* basal expression is low and is induced during bisexual mating [12], we first examined purified poly(A)-RNAs extracted from wild type, the *znf2*Δ mutant, and the *rze1*Δ mutant at the 12h and 24h time points during bisexual mating. One band that was larger than 3,000 nt and close to 4,000 nt was detected in both wild type and the *rze1*Δ mutant, but not in the *znf2*Δ mutant (Fig 6B). The size is consistent with the predicted approximately 3,600 nt-long mature transcript for *ZNF2*. The reduction in *ZNF2* transcript level in the *rze1*Δ mutant during bisexual mating was apparent at the 12h time point, but not at the 24h time point (Fig 5C and S6B Fig). This might be because the impact of *RZE1* deletion on the *ZNF2* transcript level becomes more modest at later time points, as we observed in the unisexual mating cells (S6B Fig). We also compared the *ZNF2* transcript size by northern hybridization using purified poly (A) RNAs extracted from wild type, the *znf2*Δ mutant, and the *rze1*Δ mutant at 48h during self-filamentation. We found that the size of the *ZNF2* transcript remained ~3.6kb in wild type and the *rze1*Δ mutant (Fig 6B).

Taken together, these results indicate that *ZNF2* only has one transcript isoform under the conditions we tested. Furthermore, the loss of *RZE1* does not appear to affect cryptococcal ability to process introns of the *ZNF2* transcripts.

### Disruption of *RZE1* reduces the number of *ZNF2* transcripts and altered their subcellular distribution

**Fig 7:**
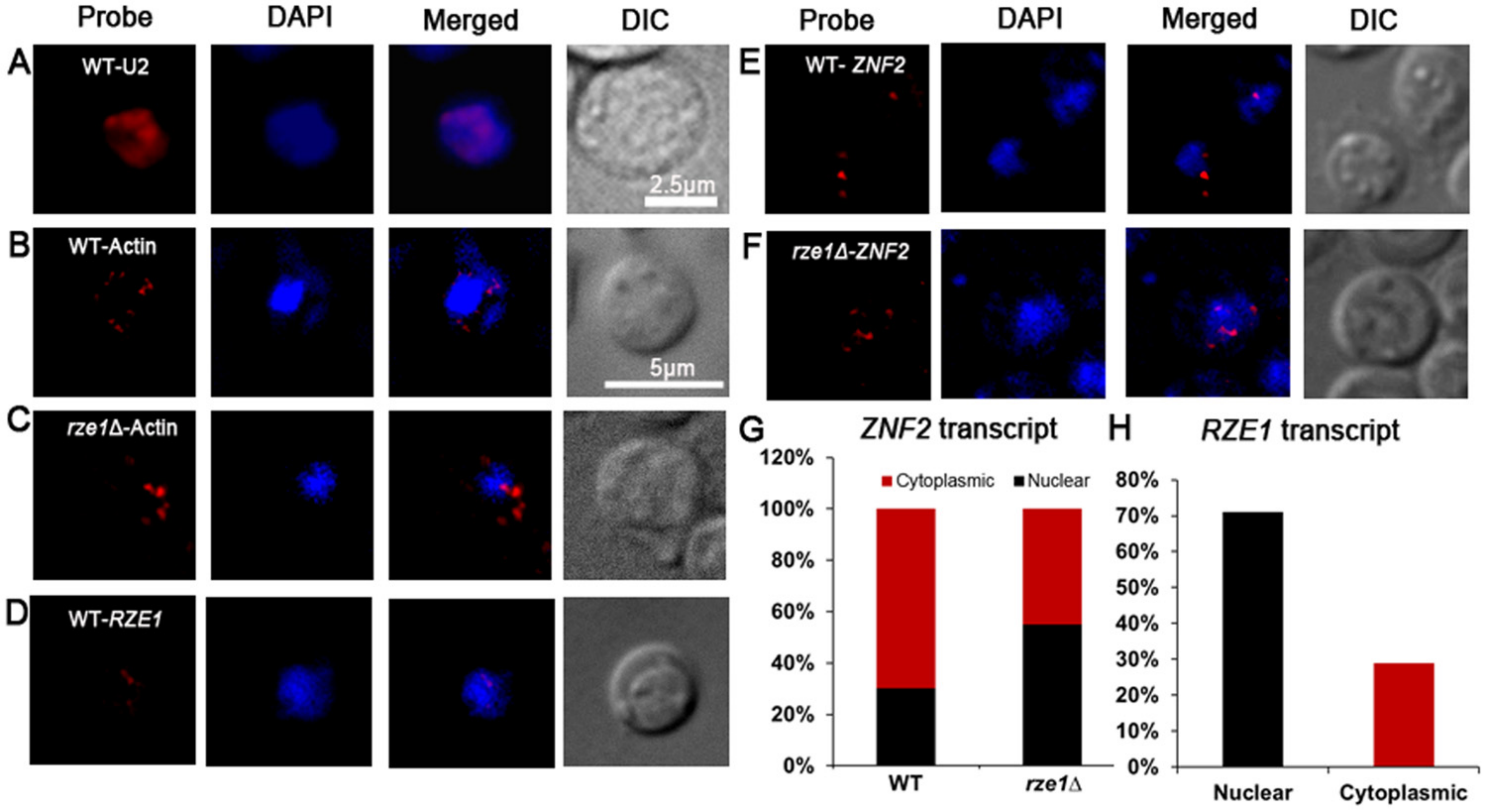
*RZE1* regulates the subcellular distribution of *ZNF2* transcripts. (A) The subcellular localization of U2 snRNA by smFISH in the wild-type strain XL280 cultured in YPD overnight. The image of the wild-type XL280 cells: bright field, Cy3 (probe of U2 snRNA or actin mRNAs), DAPI (nuclei), and Cy3 and DAPI merged. The subcellular localization of *ACT1* mRNAs in the wild type (B) and the *rze1*Δ mutant (C) cells cultured on V8 medium for 18 hours. The DIC and maximum intensity projection of z-stack images for DAPI and Cy3/*ACT1* probe channels. (D) The DIC and maximum intensity projection of z-stack images (merged for DAPI and Cy3/*RZE1* probe channels) showing the distribution of *RZE1* transcripts in the cytoplasm and the nuclei of the wild type cultured on V8 medium for 18 hours. The DIC and maximum intensity projection of z-stack images (merged for DAPI and Cy3/*ZNF2* probe channels) showing the distribution of *ZNF2* transcripts in the cytoplasm and the nuclei of the wild type (E) and the *rze1*Δ mutant (F) cultured on V8 medium for 18 hours. The *znf2*Δ mutant processed under the same conditions was used as negative control to calculate base level of signal. The nuclei were stained with DAPI. (G) The graphic comparison of *ZNF2* subcellular localization in the wild type or in the *rze1*Δ mutant. (H) The graphic representation of the subcellular distribution of *RZE1* in wild type.

We decided to test the third hypothesis: the effect of *RZE1* on the export of the *ZNF2* transcripts from the nucleus to the cytosol (Fig 4D). For this purpose, we used the technique called single molecule Fluorescent *I* *n* *S* *itu* Hybridization (smFISH), which helps visualize and quantify transcripts in the cell [47]. Unlike qPCR or RNA-seq that measure the total transcript level from the whole population, smFISH can measure the level of a particular transcript in a single cell and can be used to examine the heterogeneity in gene expression in that population. This microscopy based technique can also reveal the subcellular localization of the examined transcript. Although this technique has been used in research in other organisms (e.g. mammalian cells and model yeasts) [47–49], it has not been applied to *Cryptococcus* because of the unique challenge of making spheroplast/protoplast in this organism due to the presence of cell wall and polysaccharide capsule. Thus, we did pilot studies to optimize the experimental conditions for *Cryptococcus* using the U2 spliceosomal RNA as the positive control. We chose the U2 snRNA as the control because of its abundance in intron-rich eukaryotic cells and its exclusive nuclear localization [50]. Consistently, we found abundant U2 snRNA in *Cryptococcus* nuclei (Fig 7A).

To further establish a control of messenger RNAs that will be localized to the nucleus (for transcription) and cytosol (for translation), we chose to examine the actin transcripts (*ACT1*) in cells plated on mating media (V8 medium, 18h) (Fig 7B). We found that 62.7% of cells in wild type (n = 884) and 59.3% of cells in the *rze1*Δ mutant (n = 899) were positive for the actin probe, likely due to incomplete cell wall digestion and the consequent occlusion of the probe from the remaining cells (S2 Table). However, digestion for longer period led to visible damage in some cells and was thus undesirable for the purpose of assessment of the transcripts’ subcellular localization. Thus we decided not to extend enzymatic digestion to prevent cellular damage. We therefore assumed ~50–60% efficiency in probe penetration of cells prepared under such conditions. Among the actin transcripts examined, 70–80% of them were found in the cytosol and 20–30% in the nucleus (S2 Table). Such predominant distribution in the cytosol is expected for messenger RNAs as they primarily serve as templates for translation. Interestingly but not surprisingly, the deletion of *RZE1* did not affect the subcellular distribution of *ACT1*, with still 71% found in the cytosol in the *rze1*Δ mutant (Fig 7C and S2 Table).

We then proceeded to visualize the *ZNF2* transcripts in cryptococcal cells in the absence or the presence of *RZE1* under the filamentation-inducing condition that induces the expression of *ZNF2* (Fig 7E and 7F). The *znf2*Δ mutant cells processed under the same conditions were used as the negative control. We found that 49.18% of cells in wild type (n = 612) and 43.9% of cells in the *rze1*Δ mutant (n = 664) expressed *ZNF2* (S2 and S3 Tables.). We believe that the percentage of wild-type cells expressing the *ZNF2* gene was an under-estimation. This is because some of the wild type cells expressing higher levels of *ZNF2* were turning into hyphae under this condition. These hyphal cells were more likely to be damaged during cell collection from solid agar plates and during the fixation and digestion process. Thus, wild-type cells that likely expressed higher levels of *ZNF2* were also more likely to be excluded from the analysis. Nonetheless, as expected for an mRNA, we observed *ZNF2* transcripts in both the nucleus and the cytosol (Fig 7E and 7F). Interestingly, we observed a higher percentile of *ZNF2* transcripts localized in the nuclei in the *rze1*Δ mutant (44.7% cytoplasmic: 55.2% nuclear distribution compared to that in the wild type (69.6% cytoplasmic: 30.4% nuclear distribution) (Fig 7G and S2 and S3 Tables). The increased ratio of nucleus *versus* cytosol distribution of *ZNF2* transcripts in the *rze1*Δ mutant could be caused by a defect in exporting *ZNF2* transcripts to the cytoplasm, or alternatively increased cytoplasmic degradation of *ZNF2* transcripts. Improperly processed transcripts are known to be subject to degradation in the cytoplasm by RNA surveillance mechanisms [51]. However, we did not detect any apparent difference in intron splicing of the *ZNF2* transcripts generated in the *rze1*Δ mutant (Fig 5B). If *ZNF2* transcripts undergo increased degradation in the cytoplasm, the involvement of *RZE1* in that process must be, if any, indirect. This is because *RZE1* is functionally restricted to its native nucleus (Fig 4A). Thus, we favor the other possibility of increased nuclear retention of *ZNF2* transcripts (or reduced *ZNF2* transport) in the absence of *RZE1*. That said, although the direct effect of *RZE1* may be in the nucleus, we cannot exclude any indirect effect happening in the cytoplasm. Future investigation is warranted to further distinguish these hypotheses.

It was previously shown that functionally nuclear restricted lncRNAs could be physically confined to nuclei (as exemplified by lncRNAs MALATI and XIST [36, 37]). LncRNAs with nuclear function could also be distributed both in the cytoplasm and nuclei, although the cytoplasmic transcripts are considered non-functional as deductible from their mode of action (e.g. *FLO11* lncRNAs in *S*. *cerevisiae* [52]). We therefore decided to examine the subcellular localization of *RZE1* transcripts in the wild-type cells under a mating-inducing condition. We found that *RZE1* was predominantly localized in the nucleus (29.3% cytoplasmic: 70.6% nuclear distribution; n = 514) (Fig 7H and S2 Table). The predominant nuclear localization of *RZE1* again is consistent with the evidence presented earlier supporting *RZE1* acting as a transcript rather than as an mRNA.

## Discussion

Non-coding RNAs constitute a large part of genomes in higher eukaryotes [53, 54]. Among ncRNAs, lncRNAs are typically regulatory ncRNAs arbitrarily defined as transcripts of over 200 nt with minimum protein-coding potential [55]. The first discovered lncRNA H19 [56] was classified as a lncRNA in 1990 after the analysis of cloned gene sequence revealed its poor coding potential and its lack of observed sedimentation with the ribosomes [57]. The famous *Xist* RNA was identified by virtue of its exclusive expression by the inactivated X chromosome and its association with the X-Inactivation Center in 1991 [58–61]. *Xist* was classified as a lncRNA a year later due to lack of conserved open reading frames in its sequence and its exclusive nuclear localization [62]. Among the functionally characterized lncRNAs in higher eukaryotes, many are involved in growth, development, and differentiation [63–68]. Consequently, mis-regulation of these lncRNAs could result in tumerogenesis [69, 70], cardiovascular disorders [71, 72], or neurological diseases like Alzheimer’s and Parkinson’s [73]. Some of these lncRNAs are being exploited for the detection and treatment of cancers and lung diseases [74–76]. The mode of action by *Xist* in silencing the whole duplicated chromosome is being explored to treat diseases caused by trisomies [77]. Thus the investigation into the function of lncRNAs contributes significantly to our understanding of eukaryotic biology and diseases.

In lower eukaryotes such as fungi, however, few lncRNAs involved in the development and stress response have been characterized, mostly in the model systems such as *Saccharomyces cerevisiae* and *Schizosaccharomyces pombe* [52, 78, 79]. In *Neurospora crassa* and *Aspergillus flavus*, natural antisense transcripts (NATs) and lncRNAs are found to be differentially expressed in response to specific stimuli that induce developmental changes [80, 81]. One *N*. *crassa* lncRNA named *qrf* is experimentally shown to be important in maintaining circadian clock rhythmicity and clock resetting [82]. Although lncRNAs have been identified in phytopathogenic oomycete *Phytophthora*, and NATs in *Ustilago* are implicated in its virulence [83], none have been functionally characterized [84, 85]. Unfortunately, the prevalence or the potential functions of lncRNAs is unknown for human fungal pathogens.

Here we discovered and functionally characterized the lncRNA *RZE1*, which controls cryptococcal morphogenesis through its regulation of *ZNF2*. An ortholog of *RZE1* could not be identified outside of the *Cryptococcus* species complex by sequence similarity searches. Even within the *Cryptococcus* species complex, the level of sequence conservation of the *RZE1* gene is lower than protein-coding genes such as *ZNF2*, *GPD1*, or *ACT1*. This is not unexpected as lncRNAs are known to evolve at a faster rate and are sometimes species-specific. This, however, does raise the possibilities that *RZE1* either is confined to the *Cryptococcus* species, or it retains evolutionary conservation across different fungal species but does so without sequence conservation. In higher eukaryotes, instances of functional conservation of lncRNAs without sequence conservation have been observed [85–87]. Thus, a functional equivalent of *RZE1* in related and distant relatives of *Cryptococcus* is a possibility worth exploring. Another intriguing possibility is that in *Cryptococcus*, *RZE1* might have evolved to be a regulator dedicated to *ZNF2* and/or some of Znf2’s downstream targets. Consistently, the loss of *RZE1* does not affect the transcript level of genes CNAG_03365 and CNAG_03367 that are adjacent to *ZNF2*, reflective of *RZE1*’s selectivity towards *ZNF2* and/or its regulon. This makes *RZE1* different from some other lncRNAs that can act globally (e.g. *Xist* for the entire X chromosome [86–88]).

*RZE1* appears to act on *ZNF2* primarily through transcription regulation. There are several instances of lncRNAs in the model yeast *Saccharomyces* that regulate by turning on or off their target transcription directly or indirectly [48, 89]. In this case, the amplitude of *ZNF2* transcription induction was reduced about 2–3 folds in general in the *rze1*Δ mutant. Thus the role of *RZE1* in *ZNF2* transcriptional regulation appears to be more of modulating role than that of a switch. Given that other mutants with reduced *ZNF2* expression level only shows decreased but not blocked filamentation, it stands to reason that the regulatory function of *RZE1* goes beyond its effect on *ZNF2* transcription. Intriguingly, *RZE1* directly or indirectly regulates the distribution of *ZNF2* transcripts, although the exact mechanism of *RZE1* in regulating Znf2 activity is yet to be established.

Another enigmatic aspect of *RZE1* is its position effect. The genetic position of *RZE1* seems to have more drastic effect on Znf2 activity than *RZE1*’s expression level *per se*. For instance, the introduction of *RZE1* in the ‘*cis*’ position seems to restore filamentation to the *rze1*Δ mutant much more effectively than an ectopic copy of *RZE1* or the multi-copy *RZE1* in episomally maintained vector (Fig 1B and S2 and S5 Figs). When comparing the level of *RZE1* transcripts in the ‘*cis*’ complemented strain to the strains complemented with the site-directed mutated *RZE1* alleles in the *cis* position, there were wide variations in the transcript level (S8 Fig). Most of these strains expressed *RZE1* at a level higher than that in the wild type, yet their transcript level does not correlate with their robustness in filamentation (Fig 3C and S8 Fig). Similarly, ectopically expressing *RZE1* using the strong *CTR4* or the *GPD1* promoter in the *rze1*Δ mutant yielded poorer filamentation than the *RZE1* integrated at its native locus (S5 Fig). Collectively, these observations indicate that the physical distance between *RZE1*and *ZNF2*, and consequently their newly made transcripts, matters greatly for their functional efficiency.

While we speculate that *RZE1* specifically regulates *ZNF2*, we did not find any specific repeats or regions in *RZE1* that share sequence similarity with *ZNF2*. This suggests that their direct interaction, if it exists, is unlikely to be based on simple sequence pairing. The identification of molecules directly interacting with this lncRNA might help solve that mystery. Nonetheless, *RZE1* is the first of its kind identified in *Cryptococcus*. The *RZE1*-*ZNF2* can serve as a paradigm and a stimulus for the investigation of other regulatory lncRNAs in fungal genomes. Our preliminary transcriptional analysis of XL280 and H99 strains suggests that there are more than one thousand conserved lncRNAs in serotype D and serotype A, an equivalent of 1 lncRNA gene for every 6 protein-coding genes in *Cryptococcus*. Thus, exploring the function of these lncRNAs may give novel insights into the regulatory networks that control the morphogenesis and virulence of this organism. Such an additional layer of genetic regulation might explain the complexity of life cycle and pathogenic strategies of these “simple” eukaryotes.

## Materials and Methods

### Ethics statement

All the animal experiments were performed according to the guidelines of NIH and Texas A&M University Institutional Animal Care and Use Committee (protocol numbers: 2011–22 and 2014–0049).

### Strains and growth conditions

The strains and plasmids used in this study are listed in S4 Table. Yeast cells were grown routinely on YPD media unless otherwise specified. Mating assays were conducted on V8 agar medium in the dark at 22°C as previously described [12]. Cells collected for qPCR were grown on V8 media at 22°C in ambient air or in serum at 37°C in presence of 5% CO_2_. Transformed strains were obtained by electroporation [90] or biolistic methods [91] and they were selected on YPD with 100 μg/ml of nourseothricin (NAT) or neomycin (NEO). Strains carrying genes driven by the inducible promoter of the *CTR4* gene [32] were grown in media supplemented with 25 μM of CuSO_4_ for suppression or 200 μM of bathocuproine disulphonate (BCS) for induction [32].

### Insertional mutagenesis via agrobacterium-mediated transformation and mutant screen

*Agrobacterium tumefaciencs* strain EHA105 containing the Ti plasmid pPZP-NATcc was used for the insertional mutagenesis as described previously [12, 92]. Briefly, the bacterium was grown overnight at 22°C, washed twice with sterile water, and transferred to an induction medium containing 100μM acetosyringone and incubated for 6h. Overnight culture of *Cryptococcus* grown in liquid YPD was washed in induction medium and re-suspended to get 10^7^ cells/ml. Fungal and bacterial aliquots (200μl each) were mixed and plated on induction medium and co-cultured for 3 days at 22°C. The co-cultured cells were scraped and plated onto selective medium of YPD containing the antibiotic cefotaxime to remove *Agrobacterium* and the selective drug NAT 100μg/ml for fungal transformants. A total of 63,000 transformants were generated and screened for filamentation defect on V8 juice agar media using an Olympus SZX16 stereoscope. The colonies showing filamentation defect were further tested for intact pheromone sensing pathway by using unilateral crosses with the mating type **a** reference strain JEC20. The insertion site in selected 15 transformants with *znf2*Δ phenotype was identified using inverse PCR and sequencing as described below.

### Inverse PCR and sequencing

Genomic DNA from the selected insertion mutants having *znf2*Δ like phenotype was extracted, digested with restriction enzymes, and then self-ligated as described previously [12, 92]. Primers AI076 and AI077 were used for inverse PCR. The PCR amplicons were sequenced and the sequence was BLAST searched against the *C*. *neoformans* (JEC21) serotype D genome database at GenBank to identify the insertion site.

### In vitro phenotypic assays

Phenotypic assays were performed as described previously [93]. The strains to be tested were grown overnight in YPD. The cells were washed, adjusted to the same cell density (OD_600_ = 1.0), and serially diluted. The dilutions were plated onto appropriate media for various tests. To analyze the capacity of the strain to melanize, the original culture and the dilutions were plated onto media containing L-dihydroxyphenylalanine (L-DOPA) and incubated at 37°C in the dark. To observe capsule production, cells were plated on Dulbecco’s Modified Eagle’s medium (DMEM) (Invitrogen) and grown at 37°C under 5% CO_2._ Capsule was visualized by India ink exclusion and examined under a light microscope. For testing the ability of the fungus to grow at high temperatures and their sensitivity to UV radiation, equal number of cells were plated onto YNB agar media and grown at 37°C or exposed to 300 J/m^2^ of UV for 1, 5, and 10s. Cells were then cultured at 22°C for additional 2 days. To test the tolerance of fungal cells to osmotic and other stressors, cells were grown at 22°C for 2 days on YNB medium that was supplemented with H_2_O_2_ (21mM), calcoflour white (200 μg/ml), iron chelator-bathophenanthroline disulfonic acid (300 μg/ml), SDS (0.1%, 0.01% and 0.001%/shown in SF3), or caspofungin (16 μg/ml).

### Confrontation assay

Confrontation assays were performed as described earlier [18]. Donor cells (OD_600_ = 3) were dropped onto YPD plates 2.5 days before dropping the recipient. The recipient cells (3μl) of OD_600_ 0.8 were dropped in close proximity without touching. The colonies were observed 60h after dropping of the recipient and photographed with Olympus SZX16 stereoscope.

### Rapid Amplification of cDNA Ends (RACE) cloning

Gene Racer kit (Invitrogen, CA, USA) was used to amplify 5’ and 3’ ends of *RZE1* and *ZNF2* RNA. Full length transcript of *RZE1* was amplified using the primers indicated in S5 Table from RNA extracted from cells grown on V8 for 24h following the manufacturer’s instructions. All the 3 sets of 5’ and 3’ RACE primers yielded the same start and stop termini. The amplified bands were separated on 0.8% agarose gel and extracted using the gel purification kit (Invitrogen), cloned in TOPO 2.1 (Invitrogen) and sequenced.

### Gene manipulations

The targeted deletion of *RZE1* (excluding the first 100 bp after the transcription start) was conducted by introducing the deletion construct with the 1 kb flanks of the gene and split part of the dominant marker NAT, by electroporation or biolistic transformation as described earlier. Mutants generated were confirmed by PCR and by the genetic linkage assay using meiotic progeny dissected from genetic crosses [94]. The deletion mutant in the *MAT* **a** background was obtained by crossing the *MAT*α *rze1*Δ strains with the corresponding congenic wild type **a** strains. For complementation, *RZE1* transcript with 1kb of promoter region was amplified by PCR, digested, and inserted into the plasmid pXL1 [20]. Overexpression construct was created by amplifying the entire ORF by PCR and similarly inserting into pXL1 behind *GPD1* or *CTR4* promoters as described before [31, 32]. The *RZE1* multi-copy expression strains were generated by cloning the *RZE1* with 1kb of its own promoter into the pPM8 vector [95]. The linearized vector was introduced into the auxotrophic *rze1*Δ *ura5* strain by electroporation. The primers used for the generation of the mutants and for their analysis are listed in S5 Table.

### RNA purification, qPCR, and northern blot

RNA extraction and real-time qPCR were carried out as we previously described [20]. Briefly, total RNA was extracted using PureLink RNA mini kit (Life Technologies). DNase (Ambion) treated RNA samples were analyzed on a denaturing formaldehyde agarose gel for assessing quality and concentration. Superscript III cDNA synthesis kit (Life Technology) was used for the first strand cDNA synthesis following the manufacturer’s instructions. Constitutively expressing housekeeping gene *TEF1* was used as endogenous control for the normalization of expression of the other genes studied.

For Northern blot, total RNA was extracted from cultures on V8 medium (pH = 7.0) from the indicated strains under the conditions as described in the texts. Poly(A) tailed RNAs were purified using the PolyATtract mRNA Isolation System IV (Promega) according to the manufacture’s instruction. PolyA tailed RNAs were then separated on denaturing formaldehyde agarose gels and then transferred to nylon membrane. The Random Primers DNA Labeling System (Life technologies) was used to generate probes. Primer sequences used to make gene-specific templates are listed in S5 Table.

### RNA-seq analysis

Wild-type strain XL280 and the *rze1*Δ mutant were cultured on YPD and V8 (pH = 7) media. Cells were collected at 24, 48 and 72h from mating media and used for isolation of total RNA. Strand-specific RNA-seq was performed at the TAMU genomic facility according to the standard protocol for Illumina Genome Analyzer IIx. Sequenced reads were aligned to the XL280 reference sequence [26] using Tophat [96]. Reads that aligned uniquely to the reference sequence were considered for gene expression quantification with Cufflinks [97]. Gene expression was normalized using the DESeq package [98] in R [99]. Differential expression analysis comparing mutant to wild type was performed with using a 5% false discovery rate. RNA-Seq data is deposited at NCBI (BioProject ID PRGNA278291, accession 278291).

### Site-directed mutagenesis

To introduce single nucleotide mutations in the potential translation start sites of *RZE1*, Quick Change II XL site directed mutagenesis kit from (Agilent Technologies) was used following manufacturer’s instructions. Primers for introducing the mutations (S5 Table) were designed using the QuickChange primer design program from Agilent Technologies. *RZE1* along with 1kb of its 5’ flank were amplified by PCR and cloned into pGEMT easy vector (Promega). The entire plasmid with insert was then amplified using specific primers to change each of the ATGs to AAGs or ATAs. The modified inserts were then amplified from the plasmid using PCR with high fidelity Phusion enzyme (New England Biolabs) and used as template for overlap PCR with part of NEO marker gene to obtain a merged construct of *RZE1* with 5’ flank and part of the marker. Similar overlap PCR with 3’ flank of *RZE1* and part of NEO was used to obtain the other half of insertion construct. The two parts of insertion construct were then mixed and introduced biolistically into *Cryptococcus rze1*Δ mutant strain to replace the *rze1*Δ deletion construct. The stable transformants that showed resistance to NEO (indicative of the integration of the mutant *RZE1* allele) and sensitivity to NAT (indicative of the loss of the *rze1*Δ construct) were selected and further analyzed by PCR to confirm the replacement.

### Virulence assay using the inhalation infection model of murine cryptococcosis

Female A/J mice of 6–8 week were intranasally inoculated with 5x10^4^ cells/mouse of serotype A strains or 5x10^5^ cells/mouse of serotype D strains with 5 mice each group as we previously described [100–102]. The mice were sacrificed 10 days post inoculation. The organs from sacrificed animals were dissected and homogenized in phosphate buffered saline, serially diluted, and plated on YNB plates. After 2 days of incubation at 30°C, the CFUs were counted. One way ANOVA was used to analyze the fungal burden. All statistical analyses were performed using the Graphpad Prism 5 program and and *P* values lower than 0.05 was considered statistically significant.

### Heterokaryon assay

All strains in H99 background were grown overnight in YPD liquid medium. Cells were washed and suspended in PBS with the same cell density (OD_600_ = 1.0). Equal number of cells of each pair of strains as indicated in the figure and the text were mixed and spotted onto V8 agar pH 5.0 and incubated at 22°C in the dark for 2.5 weeks. The colony morphology was observed and photographed using an Olympus SZX16 stereo microscope.

### Measurement of fluorescence intensity

The fluorescent intensity of Znf2 tagged with mCherry was measured using the Zen Lite software from Carl Zeiss. The microscopic images taken on a Zeiss Axioplan 2 microscope were analyzed using the interactive fluorescence measurement feature of Zen. Fluorescence intensity of Znf2-mCherry from forty cells in either the wild-type background or the *rze1*Δ mutant background was measured using the software and statistically analyzed using Prism V from Graphpad.

### Single molecule FISH experiments

The smFISH experiments were performed as described before [103–105] with modifications for *Cryptococcus*. Strains either grown to exponential growth phase in YPD or the co-culture of the congenic mating pairs on V8 media were collected and fixed in 4% paraformaldehyde for 30 minutes at 22°C. The cells were digested in sphaeroplasting buffer (1M sorbitol, 10 mM EDTA, and 100 mM sodium citrate) using 100mg/ml of lyzing enzyme (Sigma) for 45 min. Sphaeroplasted cells were permeabilized after being incubated in 70% ethanol at 4°C overnight. Cells were then hybridized with TAMRA labeled *ZNF2* specific probes or ALEXA FLOUR labelled *RZE1* probes (Stellaris, Biosearch Technologies) in buffer containing 10% formamide for 16h at 37°C. Counter staining to visualize nuclei was done using 10 mg/ml of DAPI. The cells were visualized on a Zeiss Imager M2 microscope and the z-stack images were then subjected to 3D deconvolution by using AutoQuant software (Media Cybernetics). After deconvolution, the images were imported in Zen Blue software and the channels were merged and analyzed.

## Supporting Information

S1 Fig Transcript of *RZE1* identified in XL280. The potential translation start codons in the five potential open reading frames are marked.
(TIF)

S2 Fig **(A) The defect in filamentation of the *rze1*Δ mutant was partially restored by an ectopic copy of *RZE1*.** Cells of the indicated strains were grown on V8 medium for 10 days. (B) *RZE1* maintained episomally in the multi-copy vector pPM8 conferred the winkled colony phenotype (bottom image) but barely conferred any filamentation to the *rze1*Δ mutant (upper image). Cells of indicated strains were grown on V8 medium for one week.
(TIF)

S3 Fig The *rze1*Δ mutants were not different from the corresponding wild type strains in classic virulence traits like thermos-tolerance, melanization, and capsule production. There are no differences in response to cell wall stressors or oxidative agents or UV.
(TIF)

S4 Fig Ectopic complementation of the filamentation defect in the *znf2*Δ mutant using *ZNF2* with its 1kb sequences upstream of its ORF. The 1 kb sequences upstream of *ZNF2*’s ORF does not overlap with the *RZE1* transcript. This *ZNF2* construct successfully restored bisexual mating in H99 background (A and left panel in B) and self-filamentation in the *znf2*Δ mutant in XL280 background (right panel in B). The bisexual mating for strains in H99 background were cultured on V8 medium for 5 days. Strains in XL280 background were cultured on V8 medium for 3 days. The ectopically integrated *ZNF2* construct with 1 kb sequence upstream of its ORF restored the ability of the *znf2*Δ mutant to form mating hyphae during bisexual mating in H99 background in either unilateral mating (mutant x wild type) or bilateral mating (mutant x mutant).
(TIF)

S5 Fig The overexpression (P_*CTR4-2*_) of each putative open reading frame of *RZE1* does not restore filamentation in the *rze1*Δ mutant. Cells were cultured on V8 medium for 4 days.
(TIF)

S6 Fig **(A) The localization and transcription orientation of *ZNF2* and *RZE1* in XL280 wild type strain based on manual annotation of the RNA seq data in this region.** Chr: chromosome. (B) The RNA-seq reads of *ZNF2* transcripts in wild-type XL280 and the *rze1*Δ mutant cultured on V8 medium for 24 hours, 48 hours, and 72 hours.
(TIF)

S7 Fig The expression of the prominent Znf2 downstream targets *CFL1*, *FAD1*, and *FAS1* in the wild type and the *rze1*Δ mutant during self-filamentation (RNA-seq). (TIF)

S8 Fig The *RZE1* transcript level in the *rze1*Δ-*RZE1* strain, the different single nucleotide mutated *RZE1* alleles (ATG1-ATG5) integrated into its native locus in the *rze1*Δ mutant strains, and the wild type on filamentation-inducing V8 medium for 18 hours. (TIF)

S1 Table List of genes differentially expressed in the *rze1*Δ mutant and the wild type. (XLSX)

S2 Table Summary of expression and subcellular distribution of Actin, *ZNF2*, and *RZE1* transcripts. (DOCX)

S3 Table Subcellular distribution of *ZNF2* transcripts in the wild type and the *rze1*Δ mutant. (XLSX)

S4 Table Strains used in the study. (DOCX)

S5 Table Primers used in the study. (XLSX)
