# Supplementary figures and images for: The lncRNA RZE1 Controls Cryptococcal Morphological Transition
Source: PLoS Genet. 2015 Nov 20;11(11):e1005692. doi: 10.1371/journal.pgen.1005692 (PMC4654512; doi:10.1371/journal.pgen.1005692)

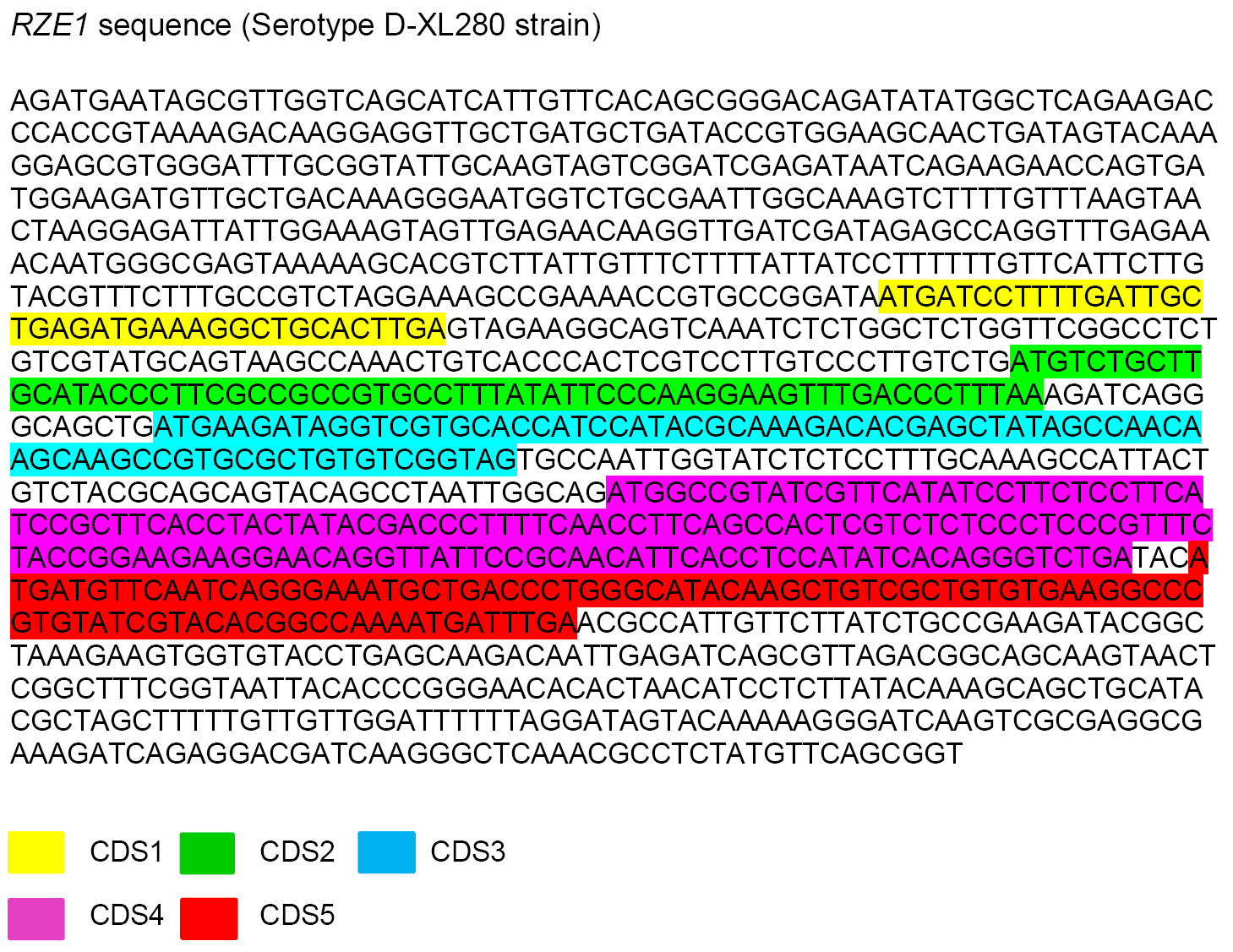

Supplement: S1 Fig — The potential translation start codons in the five potential open reading frames are marked. (TIF) [file pgen.1005692.s001.tif]

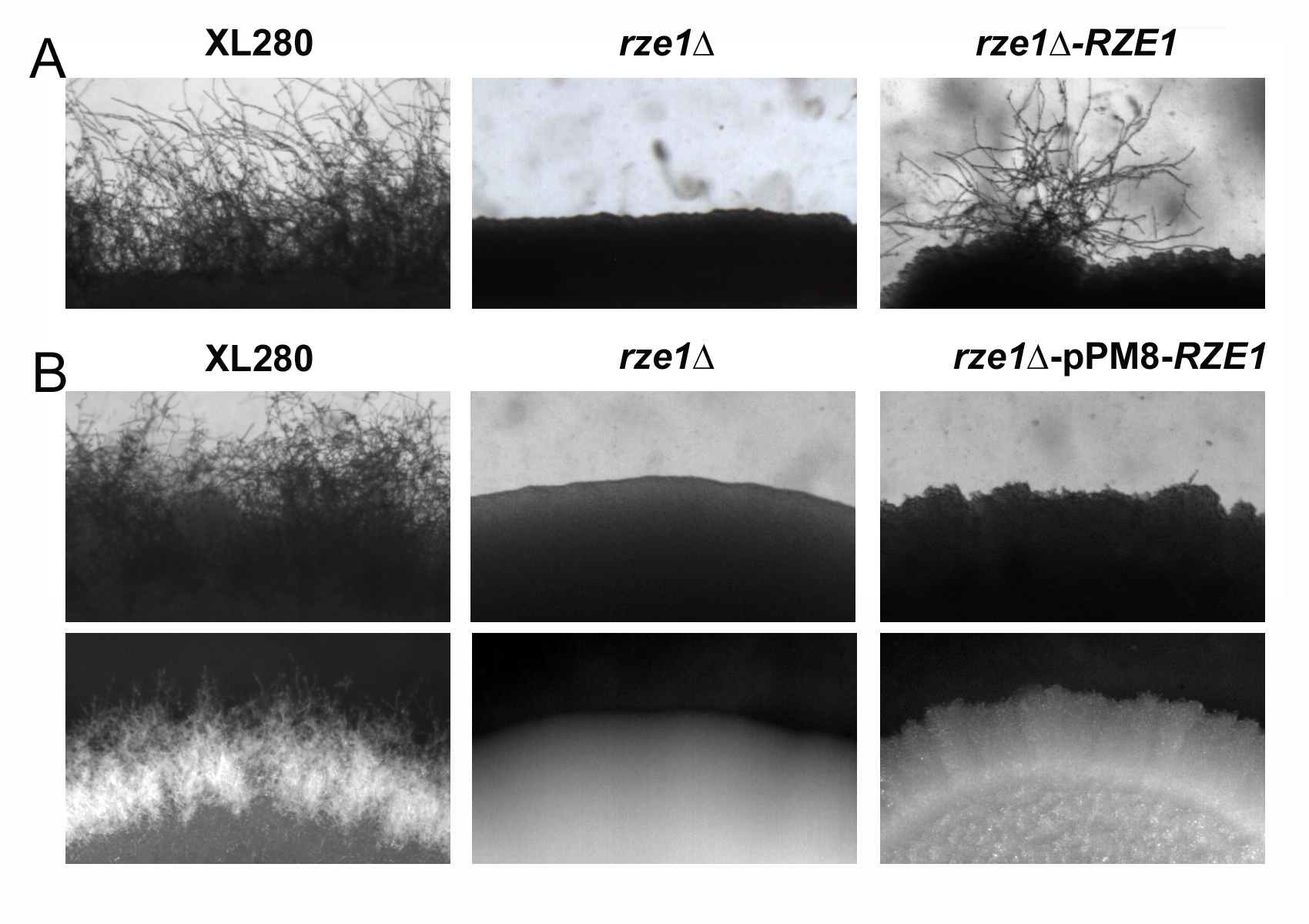

Supplement: S2 Fig — (A) The defect in filamentation of the rze1Δ mutant was partially restored by an ectopic copy of RZE1. Cells of the indicated strains were grown on V8 medium for 10 days. (B) RZE1 maintained episomally in the multi-copy vector pPM8 conferred the winkled colony phenotype (bottom image) but barely conferred any filamentation to the rze1Δ mutant (upper image). Cells of indicated strains were grown on V8 medium for one week. (TIF) [file pgen.1005692.s002.tif]

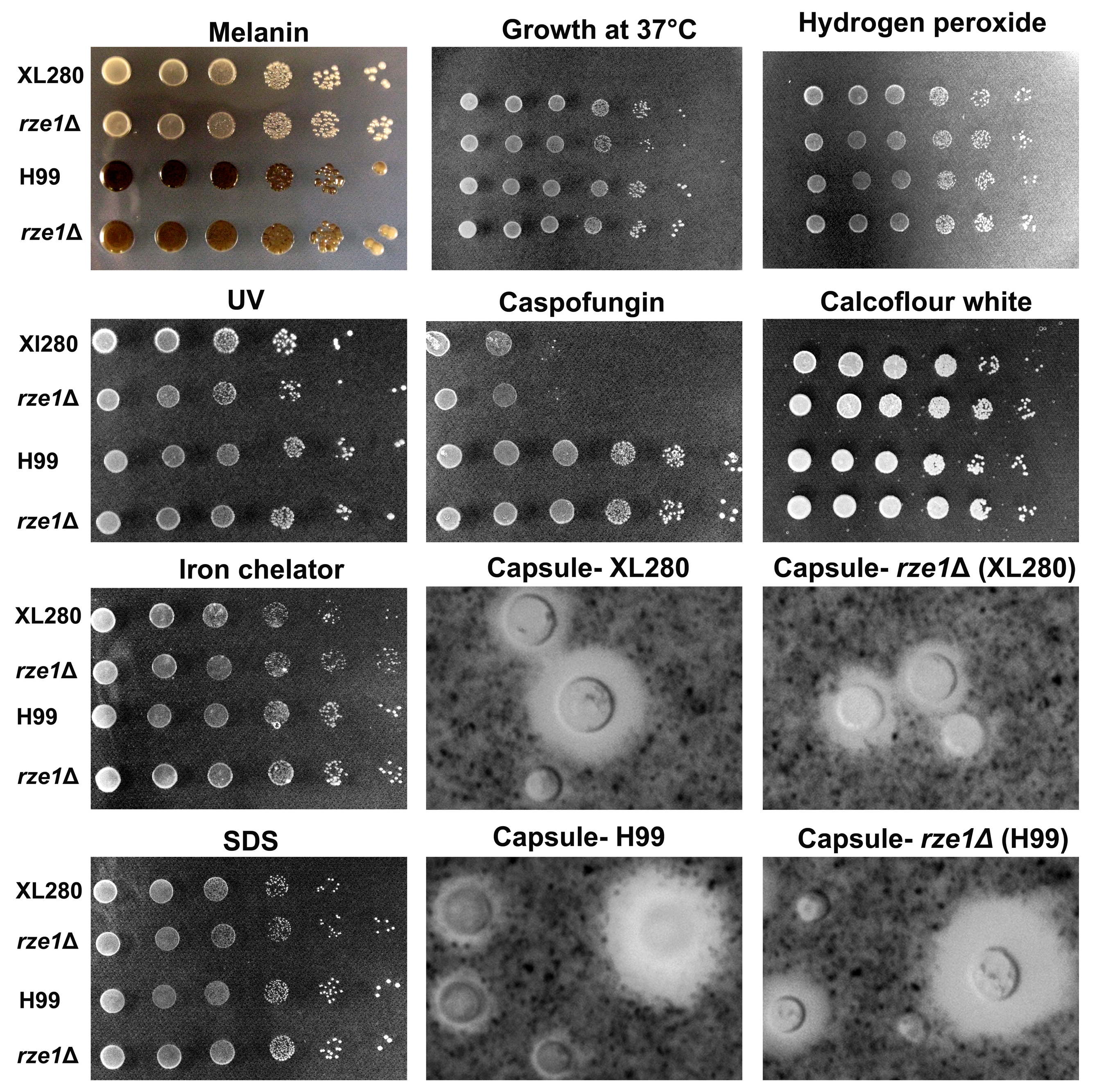

Supplement: S3 Fig — There are no differences in response to cell wall stressors or oxidative agents or UV. (TIF) [file pgen.1005692.s003.tif]

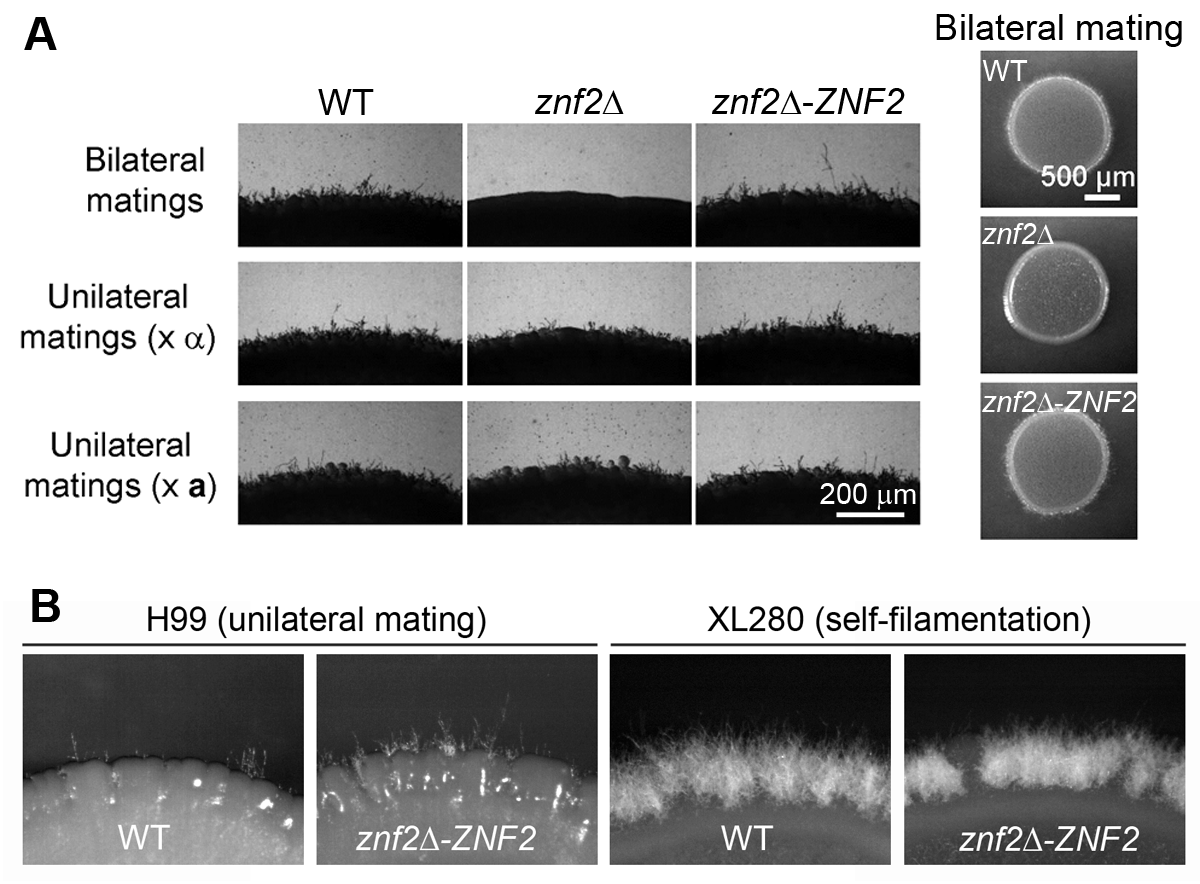

Supplement: S4 Fig — The 1 kb sequences upstream of ZNF2’s ORF does not overlap with the RZE1 transcript. This ZNF2 construct successfully restored bisexual mating in H99 background (A and left panel in B) and self-filamentation in the znf2Δ mutant in XL280 background (right panel in B). The bisexual mating for strains in H99 background were cultured on V8 medium for 5 days. Strains in XL280 background were cultured on V8 medium for 3 days. The ectopically integrated ZNF2 construct with 1 kb sequence upstream of its ORF restored the ability of the znf2Δ mutant to form mating hyphae during bisexual mating in H99 background in either unilateral mating (mutant x wild type) or bilateral mating (mutant x mutant). (TIF) [file pgen.1005692.s004.tif]

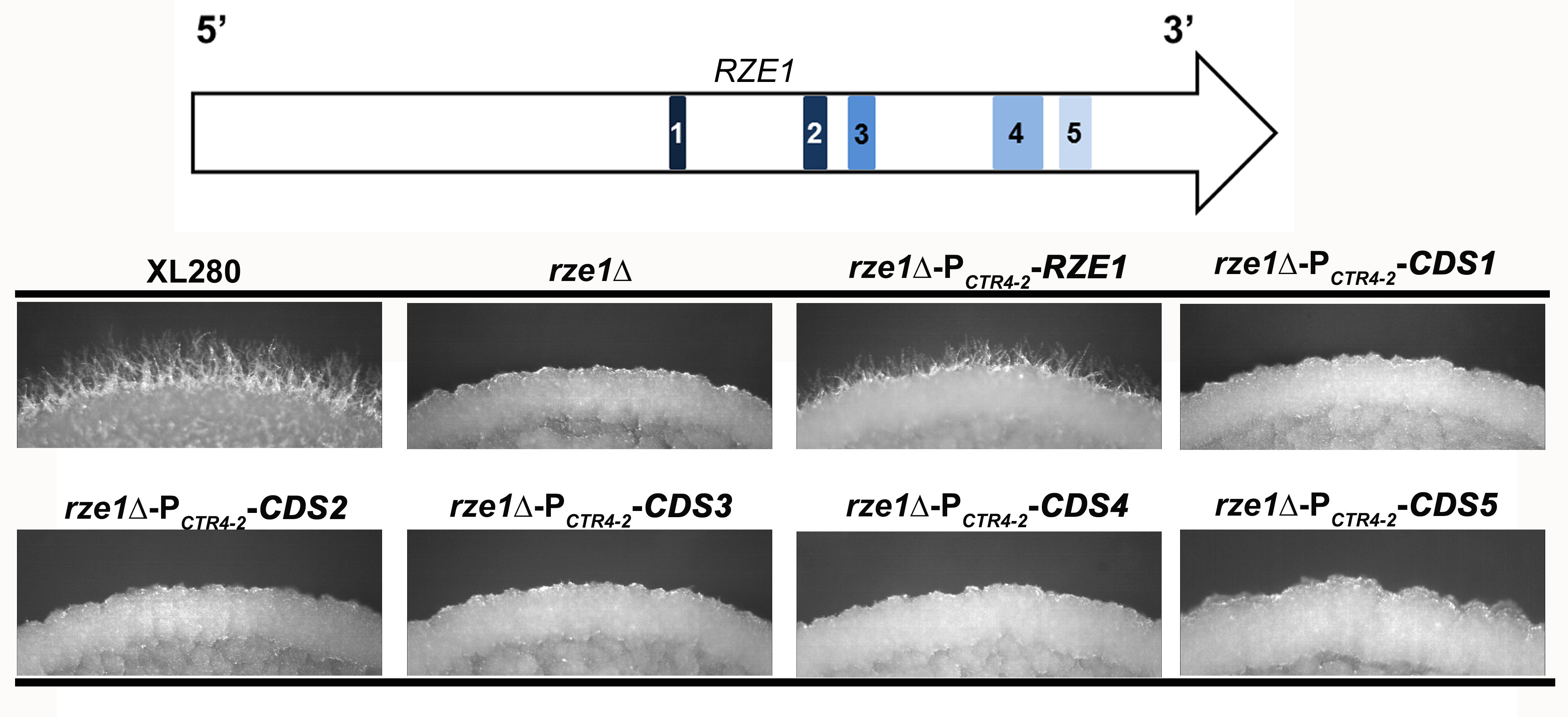

Supplement: S5 Fig — Cells were cultured on V8 medium for 4 days. (TIF) [file pgen.1005692.s005.tif]

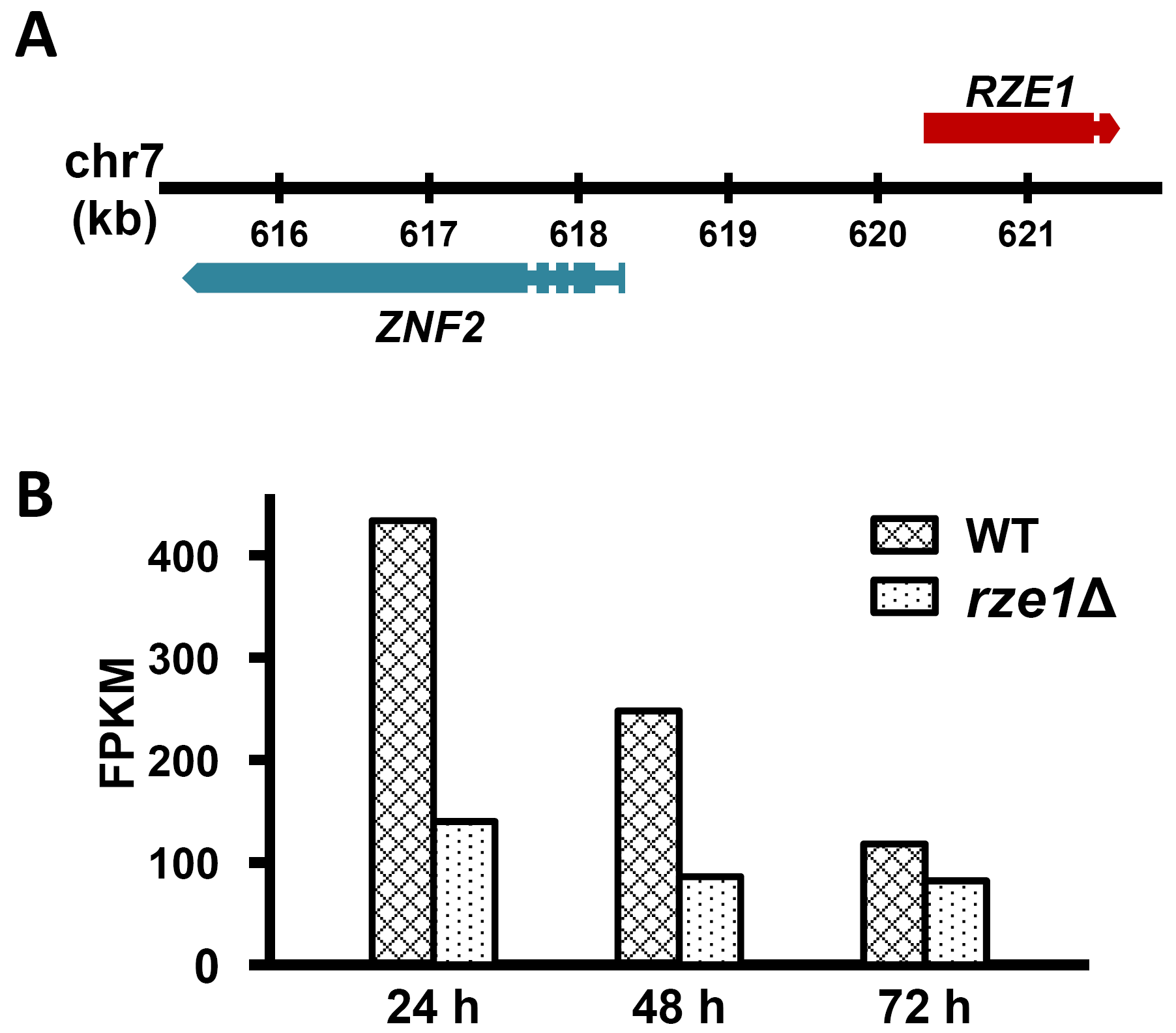

Supplement: S6 Fig — (A) The localization and transcription orientation of ZNF2 and RZE1 in XL280 wild type strain based on manual annotation of the RNA seq data in this region. Chr: chromosome. (B) The RNA-seq reads of ZNF2 transcripts in wild-type XL280 and the rze1Δ mutant cultured on V8 medium for 24 hours, 48 hours, and 72 hours. (TIF) [file pgen.1005692.s006.tif]

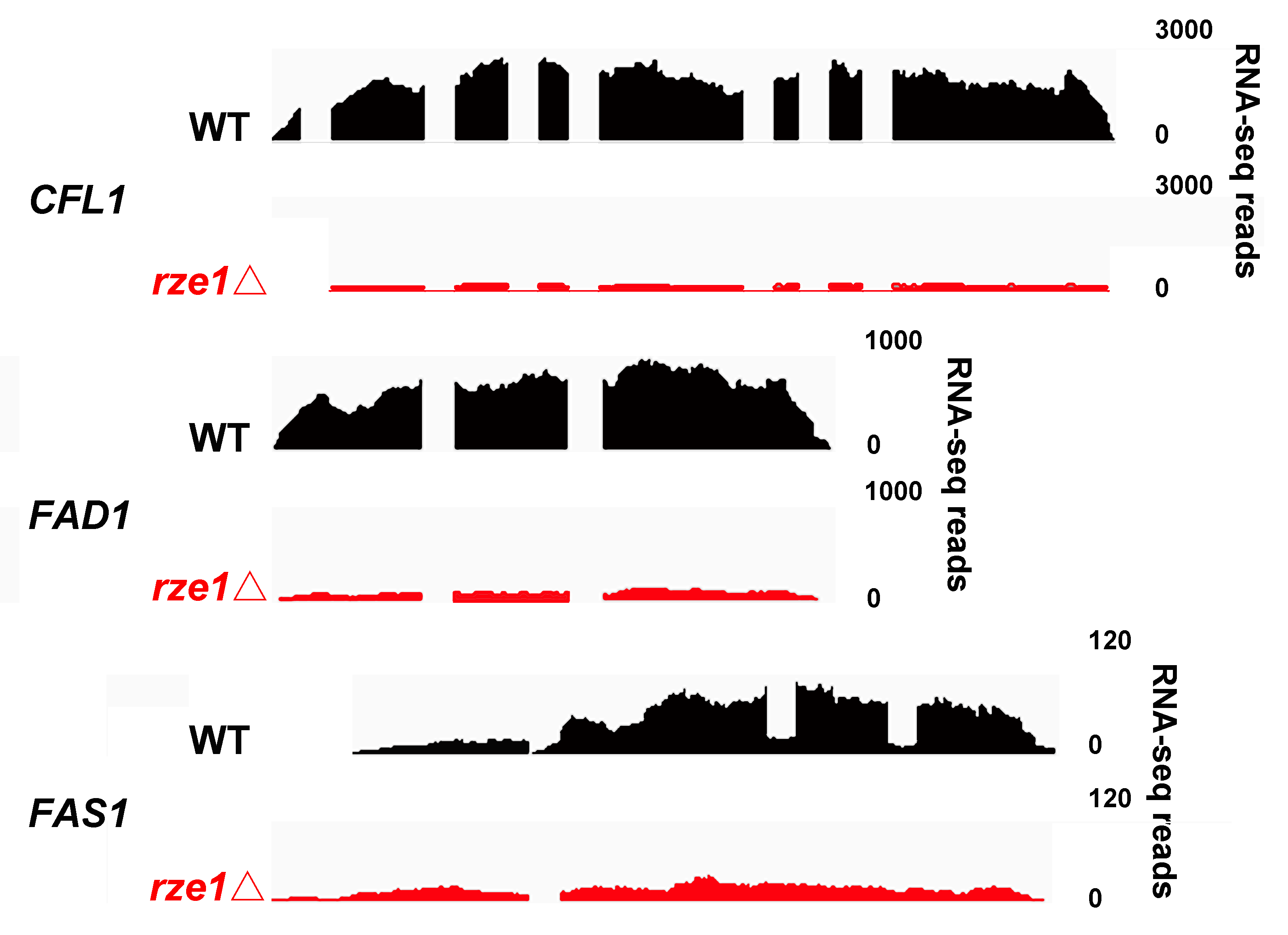

Supplement: S7 Fig — (TIF) [file pgen.1005692.s007.tif]

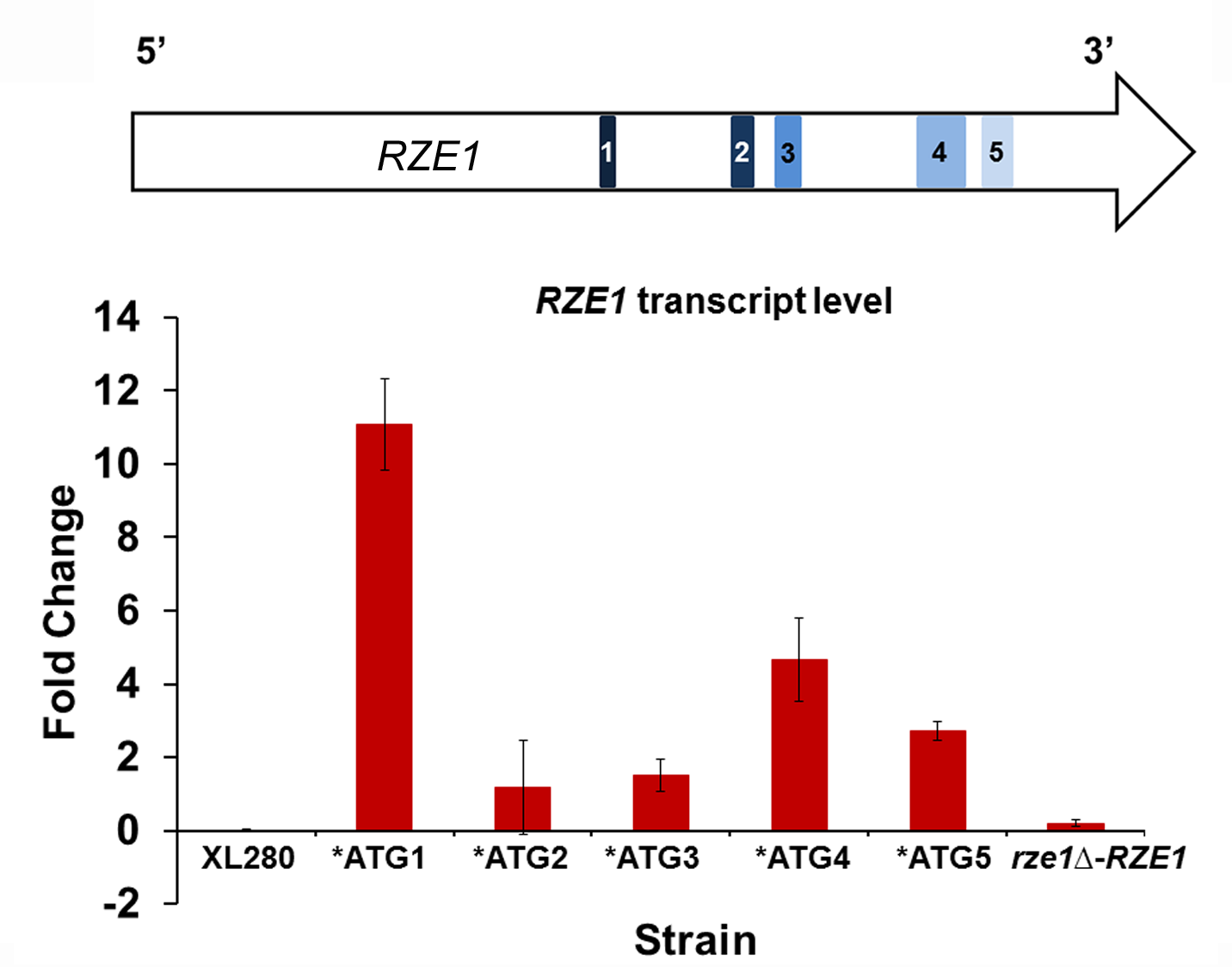

Supplement: S8 Fig — (TIF) [file pgen.1005692.s008.tif]
