# Supplementary material for: The lncRNA RZE1 Controls Cryptococcal Morphological Transition
Source: PLoS Genet. 2015 Nov 20;11(11):e1005692. doi: 10.1371/journal.pgen.1005692 (PMC4654512; doi:10.1371/journal.pgen.1005692)
Supplement: S2 Table — (DOCX) [file pgen.1005692.s010.docx]

**Table S2.** Summary of expression and subcellular distribution of *ACT1*, *ZNF2* and *RZE1* transcripts

| Actin expression | | | | | |
| --- | --- | --- | --- | --- | --- |
|  | Total no. of cells | No. of cells expressing | Avg range of transcripts/cell | Avg range in nucleus | Avg range in cytoplasm |
| XL280 | 884 | 555 (62.7%) | 1-7 | 1-2 | 1-5 |
| *rze1*∆ | 899 | 534 (59.3%) | 1-6 | 1-2 | 1-4 |
| *ZNF2* expression | | | | | |
|  | Total no. of cells | No. of cells expressing | Total no. of transcripts | Total in nucleus | Total in cytoplasm |
| XL280 | 612 | 301 (49.18%) | 654 | 199 (30.43%) | 455 (69.57%) |
| *rze1*∆ | 664 | 292 (43.9%) | 648 | 358 (55.2%) | 290 (44.7%) |
| *RZE1* expression | | | | | |
| XL280 | 514 | 134 (26.07%) | 208 | 147 (70.6%) | 61 (29.3%) |
