# Supplementary material for: The lncRNA RZE1 Controls Cryptococcal Morphological Transition
Source: PLoS Genet. 2015 Nov 20;11(11):e1005692. doi: 10.1371/journal.pgen.1005692 (PMC4654512; doi:10.1371/journal.pgen.1005692)
Supplement: S4 Table — (DOCX) [file pgen.1005692.s012.docx]

| **Strain name** | **Genotype** | **Source and comments** |
| --- | --- | --- |
| XL280α | Wild type | [[1](#_ENREF_1)] |
| AIJ18 | Wild type, Congenic pair of XL280α | [[2](#_ENREF_2)] |
| KN99α | Wild type | [[3](#_ENREF_3)] |
| KN99**a** | Wild type, Congenic pair of KN99α | [[3](#_ENREF_3)] |
| JEC20**a** | Wild type, Congenic pair of JEC21α | [[4](#_ENREF_4),[5](#_ENREF_5)] |
| LW87 | P*_GPD1_-CFL1*-NEO^r^ | [[6](#_ENREF_6)] |
| XL942^1^ | *mat2*::*NAT*^r^ | [[7](#_ENREF_7)] |
| XL1601α^2^ | *znf2*::*NEO*^r^ | [[7](#_ENREF_7)] |
| XL1637**a**^2^ | *znf2*:: *NEO*^r^ | [[7](#_ENREF_7)] |
| LW25α^1^ | *rze1*::*NAT*^r^ P*_GPD1_-ZNF2*-*NEO*^r^ | This study |
| X261α^1^ | *rze1*::Tn-*NAT*^r^ | This study |
| NC36α^1^ | *rze1*::Tn-*NAT*^r^ P*_RZE1_-RZE1*-*NEO*^r^ | This study |
| NC01α^1^ | *rze1*::*NAT*^r^ | This study |
| NC02**a**^1^ | *rze1*::*NAT*^r^ | This study |
| NC08α^1^ | *rze1*::*NAT*^r^ P*_CTR4-2_-ZNF2*-*NEO*^r^ | This study |
| NC09α^1^ | *rze1*:: *NAT*^r^ P*_GPD1_*-*RZE1*-*NEO*^r^ | This study |
| NC43α^1^ | *rze1*::*NAT*^r^ P*_CTR4-2_-RZE1*-*NEO*^r^ | This study |
| NC10α^1^ | *rze1*:: *NAT*^r^  P*_RZE1_*-*RZE1*-*NEO*^r^ | This study |
| NC12**a**^2^ | *rze1*::*NAT*^r^ | This study |
| NC13^2^ | *rze1*::*NAT*^r^ | This study |
| NC14α^2^ | *rze1*:: *NAT*^r^  P*_RZE1_*-*RZE1*-*NEO*^r^ | This study |
| NC 18 α/**a**^2^ | *rze1:: NAT*^r^  *ZNF2*-*NEO*^r^ diploid | This study |
| NC21α^2^ | *rze1*:: *NAT*^r^  *znf2*:: *NEO*^r^ | This study |
| NC23α^1^ | *rze1*::*NAT*^r^ *RZE1*ATG4-*NEO*^r^ | This study |
| NC26α^1^ | *rze1*::*NAT*^r^ *RZE1*ATG1- *NEO*^r^ | This study |
| NC31α^1^ | *rze1*::*NAT*^r^ *RZE1*ATG2- *NEO*^r^ | This study |
| XX9α^2^ | P*_CTR4-2_*-*ZNF2*::*mCherry*-*NEO*^r^ | Unpublished |
| NC40α^2^ | *rze1*::*NAT*^r^ P*_CTR4-2_*-*ZNF2*-*mCherry*-*NEO*^r^ | This study |
| NC41α^1^ | *rze1*::*NAT*^r^ *RZE1*ATG3-*NEO*^r^ | This study |
| NC42 α^1^ | *rze1*::*NAT*^r^ *RZE1*ATG5-*NEO*^r^ | This study |
| YZ01α^1^ | *rze1*::*NAT*^r^ P*_GPD1_*-*CDS1*-*NEO*^r^ | This study |
| YZ02α^1^ | *rze1*::*NAT*^r^ P*_GPD1_*-*CDS2*- *NEO*^r^ | This study |
| YZ03α^1^ | *rze1*::*NAT*^r^ P*_GPD1_*-*CDS3*-*NEO*^r^ | This study |
| YZ04α^1^ | *rze1*::*NAT*^r^ P*_GPD1_*-*CDS4*-*NEO*^r^ | This study |
| YZ05α^1^ | *rze1*::*NAT*^r^ P*_GPD1_*-*CDS5*-*NEO*^r^ | This study |
| YZ06α^1^ | *rze1*::*NAT*^r^ P*_CTR4-2_*-*CDS1*-*HYG*^r^ | This study |
| YZ07α^1^ | *rze1*::*NAT*^r^ P*_CTR4-2_*-*CDS2*-*HYG*^r^ | This study |
| YZ08α^1^ | *rze1*::*NAT*^r^ P*_CTR4-2_*-*CDS3*-*HYG*^r^ | This study |
| YZ09α^1^ | *rze1*::*NAT*^r^ P*_CTR4-2_*-*CDS4-HYG*^r^ | This study |
| YZ10α^1^ | *rze1*::*NAT*^r^:: P*_CTR4-2_*-*CDS5*- *HYG*^r^ | This study |
| NC44α^1^ | *rze1*:: *NAT*^r^  *RZE1*-*NEO*^r^ | This study |
| NC45 α^1^ | *rze1*:: *NAT*^r^  *ura5* | This study |
| NC46α^1^ | *rze1*:: *NAT*^r^  *ura5* pPM8-*RZE- URA5* | This study |
| XL904α^1^ | *znf2::NAT ZNF2-NEO* | This study |
| XL1643α^2^ | *znf2::NEO ZNF2-NAT* | This study |

Note: ^1^ strains in XL280 background; ^2^ strains in in H99 background

1. Lin X, Huang JC, Mitchell TG, Heitman J (2006) Virulence attributes and hyphal growth of *C. neoformans* are quantitative traits and the *MATα* allele enhances filamentation. PLoS Genet 2: e187.

2. Zhai B, Zhu P, Foyle D, Upadhyay S, Idnurm A, et al. (2013) Congenic strains of the filamentous form of *Cryptococcus neoformans* for studies of fungal morphogenesis and virulence. Infect Immun 81: 2626-2637.

3. Nielsen K, Cox GM, Wang P, Toffaletti DL, Perfect JR, et al. (2003) Sexual cycle of *Cryptococcus neoformans* var. *grubii* and virulence of congenic a and *α* Isolates. Infect Immun 71: 4831-4841.

4. Kwon-Chung KJ, Kozel TR, Edman JC, Polacheck I, Ellis D, et al. (1992) Recent advances in biology and immunology of *Cryptococcus neoformans*. J Med Vet Mycol 30: 133-142.

5. Heitman J, Allen B, Alspaugh JA, Kwon-Chung KJ (1999) On the origins of congenic *MAT*α and *MAT*a strains of the pathogenic yeast *Cryptococcus neoformans*. Fungal Genet Biol 28: 1-5.

6. Wang L, Zhai B, Lin X (2012) The link between morphotype transition and virulence in *Cryptococcus neoformans*. PLoS Pathog 8: e1002765.

7. Lin X, Jackson JC, Feretzaki M, Xue C, Heitman J (2010) Transcription factors Mat2 and Znf2 operate cellular circuits orchestrating opposite- and same-sex mating in *Cryptococcus neoformans*. PLoS Genet 6: e1000953.
